# The curious case of proton migration under pressure in the malonic acid and 4,4′-bi­pyridine cocrystal

**DOI:** 10.1107/S2052252524000344

**Published:** 2024-01-13

**Authors:** Ewa Patyk-Kaźmierczak, Fernando Izquierdo-Ruiz, Alvaro Lobato, Michał Kaźmierczak, Ida Moszczyńska, Anna Olejniczak, J. Manuel Recio

**Affiliations:** a Facuty of Chemistry, Adam Mickiewicz University in Poznań, Uniwersytetu Poznańskiego 8, Poznań 61-614, Poland; b MALTA-Consolider Team and Departamento de Química Física, University Complutense of Madrid, Avda. de Séneca, 2 Ciudad Universitaria, Madrid 28040, Spain; c MALTA-Consolider Team and Departamento de Química Física y Analítica, University of Oviedo, Julián Clavería n° 8, Oviedo 33006, Spain; Sun Yat-Sen University, China

**Keywords:** proton-transfer reactions, high pressure, Δp*K*_a_ rule, crystal engineering, co-crystals, organic solid-state reactions, density functional theory, molecular crystals

## Abstract

In the search for new active pharmaceutical ingredients, the precise control of the chemistry of cocrystals becomes essential. One crucial step within this chemistry is proton migration between cocrystal coformers to form a salt, usually anticipated by the empirical Δp*K*_a_ rule. Due to the effective role it plays in modifying intermolecular distances and interactions, pressure adds a new dimension to the Δp*K*_a_ rule. Still, this variable has been scarcely applied to induce proton-transfer reactions within these systems. In our study, high-pressure X-ray diffraction and Raman spectroscopy experiments, supported by DFT calculations, reveal modifications to the protonation states of the 4,4′-bi­pyridine (BIPY) and malonic acid (MA) cocrystal (BIPYMA) that allow the conversion of the cocrystal phase into ionic salt polymorphs. On compression, neutral BIPYMA and monoprotonated (BIPYH^+^MA^−^) species coexist up to 3.1 GPa, where a phase transition to a structure of *P*2_1_/*c* symmetry occurs, induced by a double proton-transfer reaction forming BIPYH_2_^2+^MA^2−^. The low-pressure *C*2/*c* phase is recovered at 2.4 GPa on decompression, leading to a 0.7 GPa hysteresis pressure range. This is one of a few studies on proton transfer in multicomponent crystals that shows how susceptible the interconversion between differently charged species is to even slight pressure changes, and how the proton transfer can be a triggering factor leading to changes in the crystal symmetry. These new data, coupled with information from previous reports on proton-transfer reactions between coformers, extend the applicability of the Δp*K*_a_ rule incorporating the pressure required to induce salt formation.

## Abstract 2

Pressure was successfully used to induce single and double proton-transfer reactions in malonic acid and the 4,4′-bi­pyridine cocrystal. After contrasting with similar literature examples, an extended correlation between the Δp*K*_a_ values of coformers and the pressure necessary to initiate proton-transfer reactions is unveiled.

## 1. Introduction

Organic cocrystals and crystalline organic salts are multicomponent crystals (Grothe *et al.*, 2016 ▸) which attract a lot of attention from the research community owing to their properties and potential applications. Organic cocrystals enable tailored optoelectronic, electronic and magnetic properties of materials (Sun *et al.*, 2018 ▸; Jiang *et al.*, 2021 ▸), whereas organic salts can exhibit ionic conductivity (Bryce, 1991 ▸; Wang *et al.*, 2022 ▸) and high proton conductivity (Xing *et al.*, 2018 ▸) when they form crystalline porous organic salts (Yu *et al.*, 2020 ▸), an ability for fast transport of polar molecules (Xu *et al.*, 2022 ▸), or can even be used for harvesting atmospheric water (Zhang, Fu *et al.*, 2022 ▸). Non-crystalline organic salts also have a wide range of applications, for example in photovoltaics (Bates & Lunt, 2017 ▸), as ionic liquids (Kaur *et al.*, 2022 ▸), or as GUMBOS (group of uniformed materials based on organic salts) and nanoGUMBOS (Tesfai *et al.*, 2009 ▸).

Both types of multicomponent crystals play an important role in the pharmaceutical industry (Putra & Uekusa, 2020 ▸). The synthesis of salts and cocrystals of active pharmaceutical ingredients (APIs) is a well established strategy for the modification of physicochemical properties of the crystal form of APIs, aimed at improving their bioavailability (Cheney *et al.*, 2010 ▸; Sigfridsson *et al.*, 2019 ▸; Li *et al.*, 2020 ▸), stability (Sigfridsson *et al.*, 2019 ▸; Liu *et al.*, 2022 ▸) and processability (Karki *et al.*, 2009 ▸; Sanphui *et al.*, 2015 ▸). In this approach, the API is paired with a compound safe for administration in humans, including other APIs already approved for use, and generally recognized as safe (GRAS; US Food and Drug Administration, 2023 ▸) compounds.

To facilitate the formation of crystalline lattices containing two chemical components, their molecules should form complementary intermolecular interactions or one should act as a hydrogen atom donor (Brønsted–Lowry acid) and the other as a hydrogen atom acceptor (Brønsted–Lowry base). For Brønsted–Lowry acid–base pairs, the difference in the acidity and basicity of the two compounds is key to determine whether a cocrystal or a salt is formed. This relationship is described by the empirical Δp*K*_a_ rule. Initially, it was based on experimental observations and stated that the formation of a salt is expected when Δp*K*_a_ (Δp*K*_a_ = p*K*_a [protonated base]_ − p*K*_a [acid]_) is higher than 2 or 3 (Stahl & Wermuth, 2002 ▸). This rule was tested against almost 6500 structures of ionized and non-ionized acid–base pairs (Cruz-Cabeza, 2012 ▸) deposited in the Cambridge Structural Database (CSD, Groom *et al.*, 2016 ▸) and more precise limits were assigned. It was established that for Δp*K*_a_ higher than 4 salts prevail, whereas for values lower than −1 cocrystals dominate. For the Δp*K*_a_ in the −1 to 4 range, a roughly 40:60 salt:cocrystal ratio is observed, with the 50:50 ratio falling at Δp*K*_a_ *ca* 1. These ratios could be modified depending on the crystal packing and molecular structure (Cruz-Cabeza *et al.*, 2022 ▸).

Still, for the APIs that are weak acids or bases, it can be hard to find an appropriate GRAS/API compound that is a strong enough counterpart to propagate hydrogen atom transfer and salt formation under ambient conditions. These molecules that are attractive for pharmaceutical applications require a change in their properties to obtain the desired characteristics of the salt compounds.

The acidity of the compound can be modified by interfering in the chemical structure or by changing the experimental protocol (Perrin *et al.*, 1981 ▸; Samuelsen *et al.*, 2019 ▸). However, altering the molecular structure can affect the medical performance of the API (Vardanyan & Hruby, 2014 ▸), or the derivative can cause severe adverse reaction in humans (Kaniwa & Saito, 2013 ▸; Shang *et al.*, 2022 ▸). In such cases, a change of other experimental parameters should be considered.

Previous studies have shown how temperature and pressure can affect the p*K*_a_ of buffer systems (Samuelsen *et al.*, 2019 ▸). The observed response of p*K*_a_ to temperature varied depending on the chemical nature of the buffer system (it was stronger for buffers containing amino groups than for those with carb­oxy­lic acid moieties). Meanwhile, pressure up to 100 MPa affected the p*K*_a_ to a limited extent. Modification of temperature and pressure facilitates proton-transfer reactions in the liquid (Isaacs *et al.*, 1977 ▸; Koifman *et al.*, 2002 ▸; Tiefenthaler *et al.*, 2020 ▸) and solid states (Martins *et al.*, 2009 ▸; Mitani *et al.*, 1988 ▸; Song & McDermott, 2001 ▸; Szafrański & Katrusiak, 2004 ▸; Bhunia *et al.*, 2010 ▸; Cai & Katrusiak, 2012 ▸; Jones *et al.*, 2012 ▸; Yu *et al.*, 2019 ▸; Tadokoro *et al.*, 2022 ▸). However, note that, especially in the context of APIs sensitive to high temperatures, heating may lead to thermal decomposition of the crystal components, which would make this method unsuitable for applications in the pharmaceutical industry. Alternatively, pressure is not as destructive to the molecular structure.

The proton-transfer-promoting action of pressure can be associated with its ability to tune the energy barrier for the reaction, linked to the decrease in the distance between proton donor and acceptor on compression (Krishtalik, 2000 ▸; Kurzydłowski, 2022 ▸). It makes pressure a perfect means to induce transformation of cocrystals into salts. However, currently, the pressure-induced proton transfer in multicomponent crystals remains an understudied phenomenon with very few examples found in the literature, such as: (i) a proton-transfer reaction cooperative with electron transfer in the quinhydrone crystal (a crystalline complex of *p*-quinone and *p*-hydro­quinone) (Mitani *et al.*, 1988 ▸); (ii) reaction in 4,4′-bipyridinium squarate (Martins *et al.*, 2009 ▸; Reetz *et al.*, 1994 ▸; Ma *et al.*, 2017 ▸); (iii) double proton-transfer in oxalic acid dihydrate form α (Casati *et al.*, 2009 ▸; Macchi *et al.*, 2010 ▸; Bhatt *et al.*, 2016 ▸); (iv) single proton transfer in cocrystals of phenazine and 2,3-di(2-pyridinyl)pyrazine with fluoranilic acid (Kumai *et al.*, 2012 ▸; Horiuchi *et al.*, 2013 ▸); (v) pressure-induced proton transfer in the cocrystal of 4-methyl­pyridine and penta­chloro­phenol (Funnell *et al.*, 2021 ▸).

Crystal structures of organic multicomponent crystals in general are rarely studied under pressure. Among high-pressure deposits of the CSD, only a small fraction (26.75%) of such crystals can be found. This subset is considerably smaller compared with the population of multicomponent crystals in the whole CSD (42.76%). Undoubtedly, to truly understand the phenomenon of a pressure-induced proton-transfer reaction between molecules of different chemical compounds in the solid state, further studies of cocrystals and solvates structurally capable to undergo such process should be carried out.

In this work we report results on the effect of pressure on proton migration in a model cocrystal (BIPYMA), built of 4,4′-bi­pyridine (BIPY) and malonic acid (MA). The difference in the p*K*_a_ of BIPY and malonic acid (MA) is 1.99 and −2.36 for the transfer of the first and second proton (Scheme 1; MA and BIPY acid–base pair after first and second proton transfer), respectively, calculated based on the literature p*K*_a_ values (Ulstrup *et al.*, 1969 ▸; Khalil *et al.*, 2013 ▸). It was previously confirmed by X-ray diffraction (XRD) and nuclear quadrupole resonance techniques (Pedireddi *et al.*, 1998 ▸; Seliger & Žagar, 2014 ▸) that, under ambient conditions, they form a cocrystal of *C*2/*c* symmetry, with half the MA and half the BIPY molecules in the asymmetric part of the unit cell. 
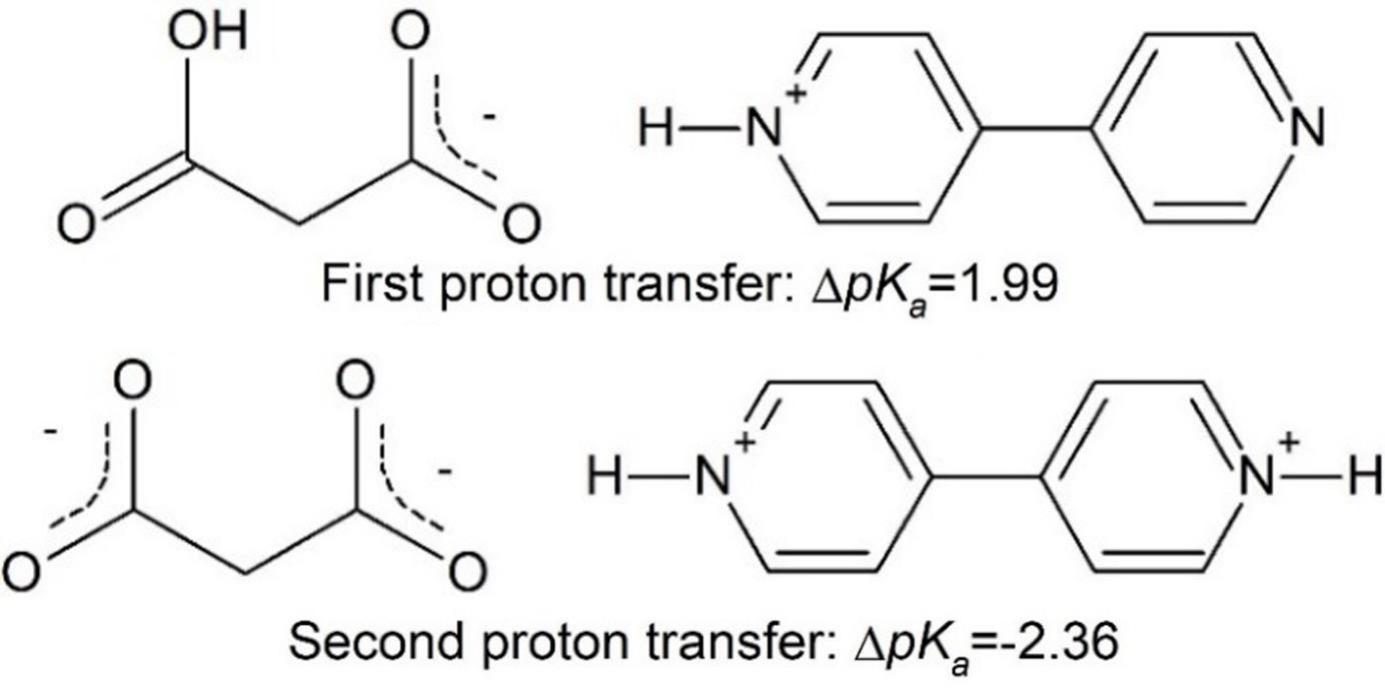


In this study we aim to answer following three questions: (i) Can pressure induce proton-transfer reactions in BIPYMA? In the case of a positive answer, (ii) which is the pressure needed for the reaction to take place? (iii) Will pressure induce a single or double proton-transfer reaction? An additional objective is to add new data on the pressure effects in cocrystals. To the best of our knowledge, our results are among very few research reports on this phenomenon. After comparison with the available examples reported in the literature, we provide new insights and understanding of proton-transfer reactions in the solid state.

## 2. Experimental methods

### 2.1. Cocrystal synthesis

4,4′-Bi­pyridine (91.54 mg) and malonic acid (51.50 mg) were dissolved in 8 ml EtOH:DMSO solution (1:1 *v*/*v* ratio) and left for slow evaporation (at room temperature, in a vial covered with a parafilm with a few needle-size holes in it) to yield colourless, needle crystals.

### 2.2. X-ray diffraction experiments under ambient conditions

X-ray diffraction data for a single crystal of BIPYMA were collected using a four-circle SuperNova single-crystal diffractometer, equipped with a Cu X-ray tube (λ = 1.54178 Å) and Atlas detector. The program *CrysAlisPro* (Rigaku Oxford Diffaction, 2019 ▸) was used for data collection.

### 2.3. Single-crystal high-pressure X-ray diffraction experiments

A single crystal of BIPYMA was mounted in One20 DAC (diamond anvil cell) from Almax Easylab (opening angle of 120°), using a steel gasket (0.15 mm thick) with a 0.43 mm opening, and covered with Daphne oil 7575 as a hydro­static medium. The crystal position was fixed with a cellulose fibre, and a ruby chip was placed in the DAC chamber to enable pressure measurement. The pressure inside the DAC was determined using the ruby-fluorescence method (Piermarini *et al.*, 1975 ▸) with a Photon Control Inc. spectrometer (affording accuracy of about 0.02 GPa). The crystal of BIPYMA was compressed isothermally to the highest pressure of 3.33 (2) GPa. The pressure was increased, both gradually and rapidly, and data were collected at 0.25 (2), 0.66 (2), 1.03 (2), 1.33 (2), 1.83 (2), 1.95 (2), 2.19 (2), 2.47 (2), 3.04 (2) and 3.33 (2) GPa for the compressed sample [with crystals under 0.25–2.47 (2) GPa measured in one series, and at 3.04 and 3.33 GPa compressed directly from ambient conditions]; and at 2.76 (2), 2.10 (2), 1.74 (2), 1.54 (2), 0.87 (2), 0.71 (2), 0.48 (2) and 0.14 (2) GPa for a decompressed crystal [to 2.76 (2) GPa decompressed from 3.33 (2) GPa, and in the 2.10–0.14 (2) GPa range decompressed from 2.47 GPa]; see Tables S1–S3 and Figs. S1–S3 of the supporting information. Use of a DAC of the wide opening angle allowed us to collect data having an average completeness in the 45–55% (structures up to 2.47 GPa) and 36–40% (structures above 2.47 GPa) ranges. Additionally, unit-cell parameters were measured for a sample crystal compressed/decompressed in the Merrill–Bassett DAC (Merrill & Bassett, 1974 ▸), prepared in the same manner as the One20 DAC in the 1.13–3.60 (2) GPa range (Table S2).

X-ray diffraction data were collected with either a New Xcalibur or an Xcalibur four-circle diffractometer, equipped with an Mo X-ray tube (λ = 0.71073 Å) and an EosS2 or EOS CCD detector, respectively. *CrysAlisPro* was used for data collection.

### 2.4. Crystal structure solution and refinement

*CrysAlisPro* was used for **UB** matrix determination, data reduction and absorption correction. High-pressure crystal structures were solved by intrinsic methods using *ShelXT* (Sheldrick, 2015*b* ▸) and refined with the least-squares method in *ShelXL* (Sheldrick, 2015*a* ▸), using *Olex2* (Dolomanov *et al.*, 2009 ▸) as an interface. The ambient-conditions BIPYMA structure was solved using intrinsic phasing [with *ShelXT* (Sheldrick, 2015*b* ▸)] and was non-spherically refined with *NoSpherA2* (Kleemiss *et al.*, 2021 ▸; Midgley *et al.*, 2021 ▸) implemented in *Olex2*, using *Orca* (version 5.0; Neese, 2022 ▸) with the PBE method, *cc-pVTZ* basis set and multiplicity of 2. For the structure at 0.1 MPa, the position of hydrogen atoms was assigned based on electron density, and refined using anisotropic thermal factors. Due to the limited completeness (below 55%) and quality of the high-pressure data, the hydrogen atoms in the high-pressure structures were located at idealized positions based on the molecular geometry and assigned isotropic thermal parameters depending on the equivalent displacement parameters of their carriers. The position of the acidic hydrogen atom (on the MA oxygen versus the BIPY nitro­gen atom) was assigned based on the geometry of the carb­oxy­lic group (the length of the C=O and C—O bonds) and DFT calculations (for additional details see the supporting information). Crystal structures were deposited with the Cambridge Crystallographic Data Centre (CCDC: 2279875–2279893) and can be accessed free of charge by filling out an online form at https://www.ccdc.cam.ac.uk/structures/. Crystallographic details for all structures are listed in Table S1.

### 2.5. Raman experiments

A powder sample of BIPYMA was loaded into a membrane Merrill–Bassett DAC equipped in class IIa diamonds (alongside a ruby chip and Dahpne 7575 oil). An in-house built Raman spectrometer equipped with a multichannel detector from Hamamatsu Photonics and an M255 laser from Solar Laser Systems was used for spectra collection. Data were collected and processed using the program *SpectroLab*. Data were collected in two series, for a sample compressed gradually from ambient pressure to 3.51 GPa (Fig. S4), and rapidly to 2.77, 2.92, 2.99, 3.11, 3.24, 3.31, 3.41 and 3.51 GPa, with decompression to ambient pressure after each step (Fig. S5).

### 2.6. DFT calculations

DFT calculations under static conditions have been performed using the Vienna ab *initio* Simulation Package (*VASP*; Kresse & Furthmüller, 1996 ▸) and the projector augmented wave (PAW; Blöchl, 1994 ▸) method. We use the Perdew–Burke exchange correlation functional (Perdew *et al.*, 1996 ▸) and the Grimme D3 dispersion correction with the Becke–Johnson damping function (Grimme *et al.*, 2010 ▸). A planewave cutoff of 400 eV was chosen with a Gamma centred **k**-point mesh with a resolution of 0.25 Å^−1^. The valence active space for C, N, O and H atoms was selected as 4, 5, 6 and 1 electron(s), respectively. Vibrational properties at the gamma point were computed using the finite difference method implemented in the *PHONOPY* package (Togo *et al.*, 2023 ▸). Raman intensities were computed using the *SpectroscoPY* code (Skelton *et al.*, 2017 ▸). Energy and forces convergence thresholds were set at 10^−9^ eV and 0.005 eV Å^−1^, respectively. For further details see the supporting information.

### 2.7. CSD data mining

The Cambridge Structural Database (version 5.43; November 2022) was searched for entries containing more than one chemical unit using *ConQuest* (Bruno *et al.*, 2002 ▸) and a high-pressure subset, with polymeric structures excluded. The results (840 high-pressure deposits from 193 REFCODE families) were manually revised and the final subset of deposits was reduced by omitting structures of iron carbonate (reduction by 38 deposits in total, across 11 REFCODE families).

## 3. Results and discussion

### 3.1. Crystal symmetry and phase transition

**Figure 1:**
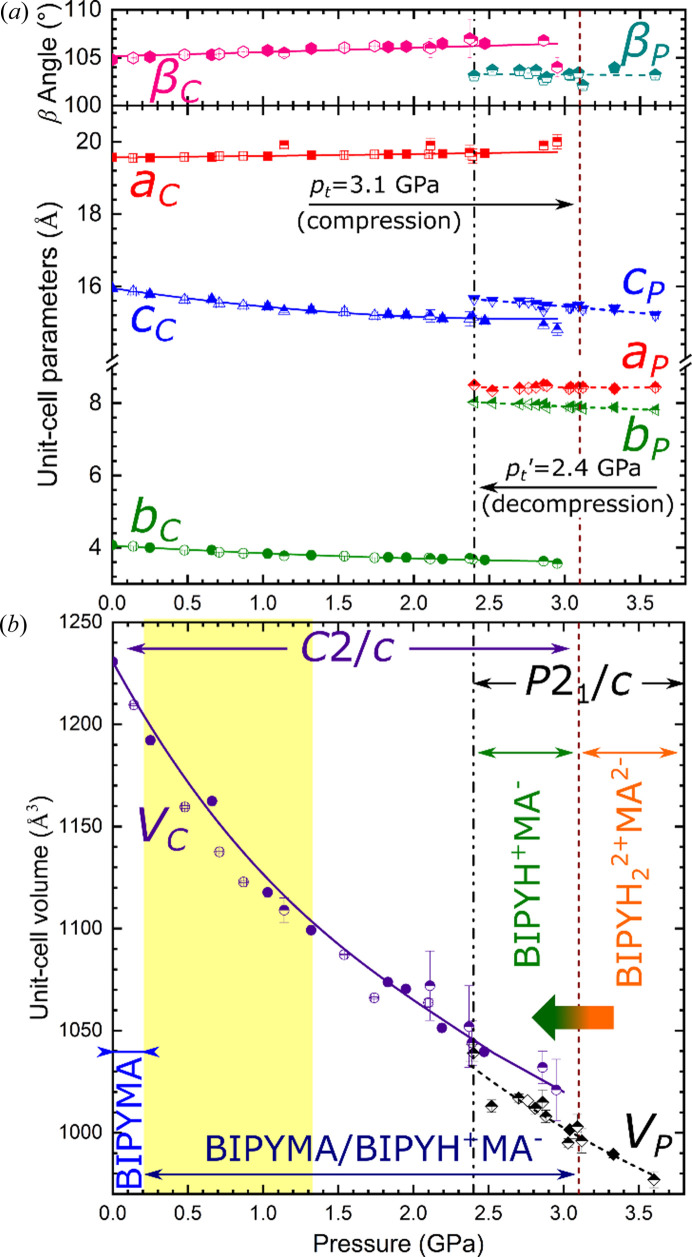
Pressure dependence of the (*a*) unit-cell parameters and (*b*) volume. Full and empty symbols represent data for the compressed and decompressed crystal (where crystal structure was solved and refined), respectively. Data for the compressed/decompressed crystals where only unit-cell parameters were measured are marked with half-empty symbols (Table S2). Horizontal arrows in panel (*b*) of the figure mark pressure regions where each form of the crystal exists and pressure regions where *C*2/*c* and *P*2_1_/*c* phases were observed (in purple and black, respectively). Orange–green gradient arrow marks the direction of the transformation from 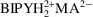 to BIPYH^+^MA^−^ in the *P*2_1_/*c* phase. Vertical dashed and dashed-dotted lines mark the transition pressures *p*_t_ and *p*_t_′ between the *C*2/*c* and *P*2_1_/*c* phases on compression and decompression, respectively. Yellow highlight marks the pressure region where the BIPYMA/BIPYH^+^MA^−^ interconversion can occur. The lines fitted to experimental points are guides to the eye only. Subscripts *C* and *P* stand for *C*2/*c* and *P*2_1_/*c*, respectively.

The series of XRD experiments, where crystalline samples of BIPYMA were compressed and decompressed, have revealed that the *C*2/*c* symmetry of the crystal is preserved on compression up to around 3.1 GPa, while above this pressure a lowering of the crystal symmetry to *P*2_1_/*c* is observed. This symmetry change is accompanied by a drop in the unit-cell volume of *ca* 20 Å^3^ (∼2%) and significant changes in the length of the unit-cell parameters (Fig. 1 ▸, Table S1). The changes during the phase transition are also captured by DFT calculations. There is a good agreement in both the volume collapse (∼3%) of the unit cell and the modification of the lattice parameters across the transition (see Figs. 1 ▸ and 2 ▸). In the measured Raman spectra, the main active modes (Fig. S6) show changes in the frequency–pressure slopes that also evidence a phase transition at 2.9 GPa, in good agreement with the experimental XRD data. The compression and phase transition also affect the crystal morphology (Figs. S2 and S3, and Movie S1 of the supporting information). The *P*2_1_/*c* phase can be preserved on decompression down to a pressure of *ca* 2.4 GPa, as confirmed by the measurement of lattice constants (Tables S1 and S2). In summary, the phase transition shows a hysteresis pressure range of about 0.7 GPa at the temperature of the experiment.

**Figure 2:**
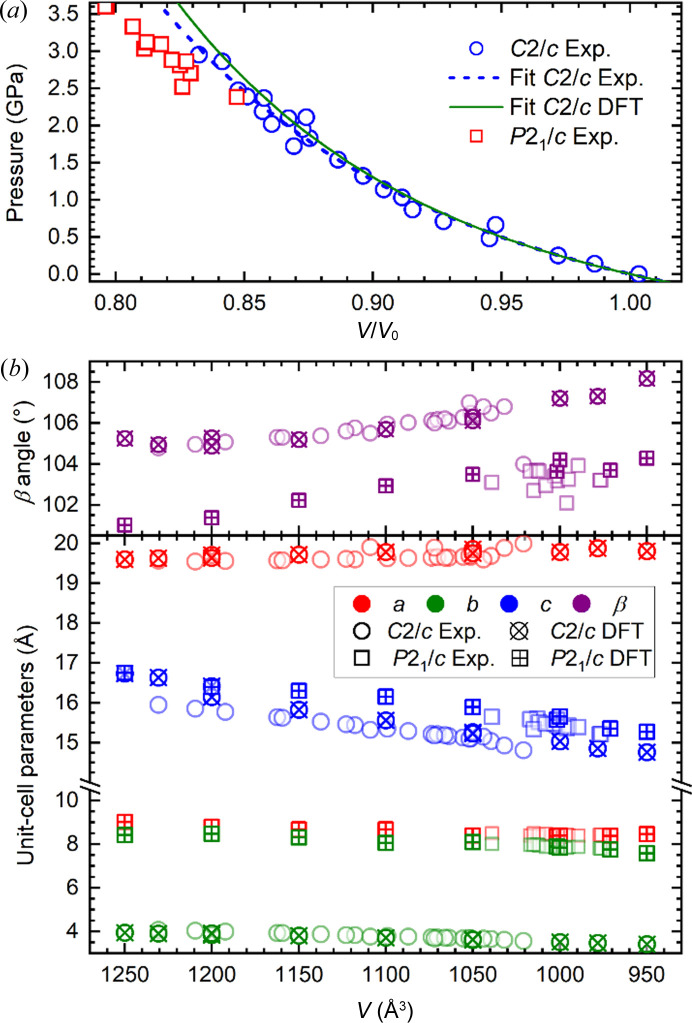
(*a*) Experimental and calculated EOS for the *C*2/*c* and *P*2_1_/*c* phases. For the *P*2_1_/*c* phase data points, *V*_0_ is assumed to be the same as for the *C*2/*c* phase. (*b*) Lattice parameters for the *P*2_1_/*c* phase (squares) and the *C*2/*c* phase (circles) obtained experimentally (empty symbols) and from DFT simulations (crossed symbols).

Experimental data are also in overall good agreement with the computed lattice and equations of state (EOS) parameters for the *C*2/*c* phase, see Fig. 2 ▸. For the *P*2_1_/*c* phase, the comparison of the EOS parameters is more difficult to carry out due to the scattering in the experimental data, and the lack of a zero-pressure volume value. A Murnaghan EOS is fitted to the experimental *P*–*V* data of the *C*2/*c* phase, yielding the following parameters: *V*_0_ = 1227 Å^3^, *B*_0_ = 8.05 GPa, *B*′_0_ = 7.1. The computed EOS with the data obtained from the DFT simulations agree very well: *V*_0_ = 1185 Å^3^, *B*_0_ = 7.89 GPa, *B*′_0_ = 8.0. For the lattice parameters, the comparison between experiment and simulation is also in good agreement. The largest deviation occurs for the lattice parameter *c* of the *C*2/*c* phase (*ca* 3%) mainly due to its strong dependence with the β angle of the monoclinic cell, which is the most sensitive parameter to optimize.

### 3.2. The pressure-induced proton transfer

**Figure 3:**
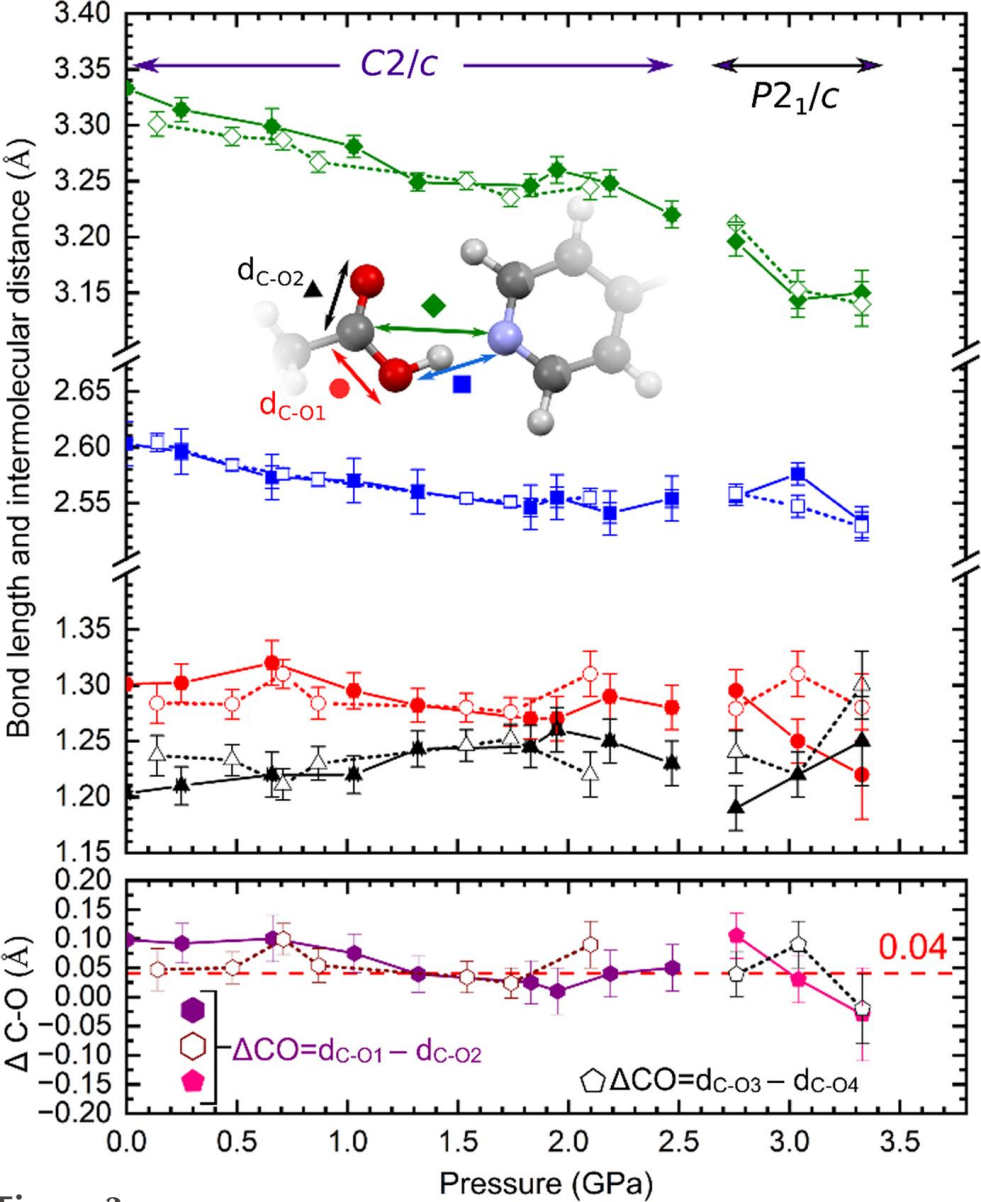
Pressure dependence of the C=O and C—O bond lengths (black triangles and red circles, respectively), as well as the O⋯N and C⋯N distances (blue squares and green diamonds, respectively). Full symbols joined with solid lines mark the compression series for the *C*2/*c* phase and the data for the carb­oxy­lic group C12(=O2)O1H of the *P*2_1_/*c* phase. Open symbols connected by dotted lines mark the decompression series for the *C*2/*c* phase and data for the carb­oxy­lic group C14(=O4)O3H of the *P*2_1_/*c* phase. The lower graph shows the difference in the length of carbon–oxygen bonds of the carb­oxy­lic group, with a dashed red horizontal line marking the difference limit of 0.04 Å. All values were calculated using *ShelXL* (Sheldrick, 2015*a* ▸) matrices. BIPY and MA moieties are related by the following symmetry codes: (i) C—O1H⋯N1*^x^* ^, *y*, *z*^; (ii) C—O3H⋯N2^1 − *x*, 1/2 − *y*, −1/2 + *z*^.

Analysis of the geometry of the carb­oxy­lic group of MA on compression up to 2.5 GPa suggests that, despite no change in the crystal symmetry, the proton-transfer reaction does occur. When the crystal is compressed, starting at 1.3 GPa, the trend points toward a shortening of the C—OH bond accompanied by an elongation of the C=O bond (Fig. 3 ▸), although the lack of data completeness (common for high-pressure experiments, especially for low-symmetry samples) limits the conclusions that can be obtained from this analysis. Note that all data points should not be analysed jointly when it comes to trends visible in them. For the *C*2/*c* phase two series (for compressed and decompressed sample crystals, marked in Fig. 3 ▸ with solid and dashed lines, respectively) should be distinguished. In such cases, when compression of the sample crystal is exclusively analysed, the increase in the length of the double C=O bond and the decrease for the single C—O bonds are more visible. The decompression series can be more biased as some effect might show hysteresis on decompression and also the sample crystal becomes more exhausted with each experimental step. Fortunately, results from the DFT calculations clearly display a reduction of the single C—O bond and an elongation of the double C=O bond on compression, confirming experimental expectations. These bond length changes are linked to the proton transfer from MA to BIPY. Our calculations corroborate that this process depends on the pressure/volume of the unit cell (Figs. S8 and S9).

Thus, at volumes of 1300 Å^3^, for both the *C*2/*c* and the *P*2_1_/*c* space groups, molecules remain in their neutral form (*i.e.* no proton has been transferred). On compression, calculations show a proton transfer between the MA and the BIPY molecules in both space groups. This behaviour occurs in the volume range 1200–1100 Å^3^ (corresponding to experimental pressures between 0.2 and 1.3 GPa) and implies that both the neutral and the monoprotonated species can coexist. In other words, the energetic difference between the two systems is almost independent of the space group (see Fig. S11 and Tables S4–S5). The energy barrier for the proton transfer in the *C*2/*c* space group is also predicted to be extremely low (below 1.6 kJ mol^−1^ at 1150 Å^3^), which means complete activation at room temperature (Fig. S12).

Therefore, it appears that the first proton transfer occurs at low pressures and generates a structure containing a mixture of neutral and singly charged BIPY. Since structures with neutral and monoprotonated BIPY entities are energetically similar, it is to be expected that hydrogen atoms in the X-ray diffraction analysis show partial occupations in the stability pressure range of the *C*2/*c* form, as indeed observed in the crystal structure refinement. For structures in the 0.2–2.5 GPa pressure range, when the difference in the length of carbon–oxygen bonds is not significant (lower than 0.04 Å), the position of the hydrogen atom should be split between the carb­oxy­lic group and the pyridine ring, reflecting the transfer to the BIPY molecule in some parts of the crystal structure (for detailed information of the acidic proton refinement, see the supporting information).

The transition to the *P*2_1_/*c* phase occurring above 3.1 GPa can be associated with the double proton transfer from MA to BIPY. This association is corroborated by our DFT calculations, where only the doubly protonated BIPY structure is observed under these conditions. Accordingly, the structural changes and the two proton migrations across the transition can be related in the following manner. As a result of the proton transfer, repulsion between positively charged 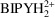 ions, stacked closely to each other in the crystal structure, arises. This triggers molecular rearrangement, resulting in breaking of the symmetry. The transition leads to the increase of *Z*′ (from 0.5 to 1), with the asymmetric part of the unit cell comprising one MA^2−^ anion and one 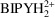 cation (compared with half an MA molecule/ion, lying on a special position of a twofold axis symmetry, and half an BIPY molecule/ion, at the special position of a symmetry of a centre of inversion, in the *C*2/*c* phase).

**Figure 4:**
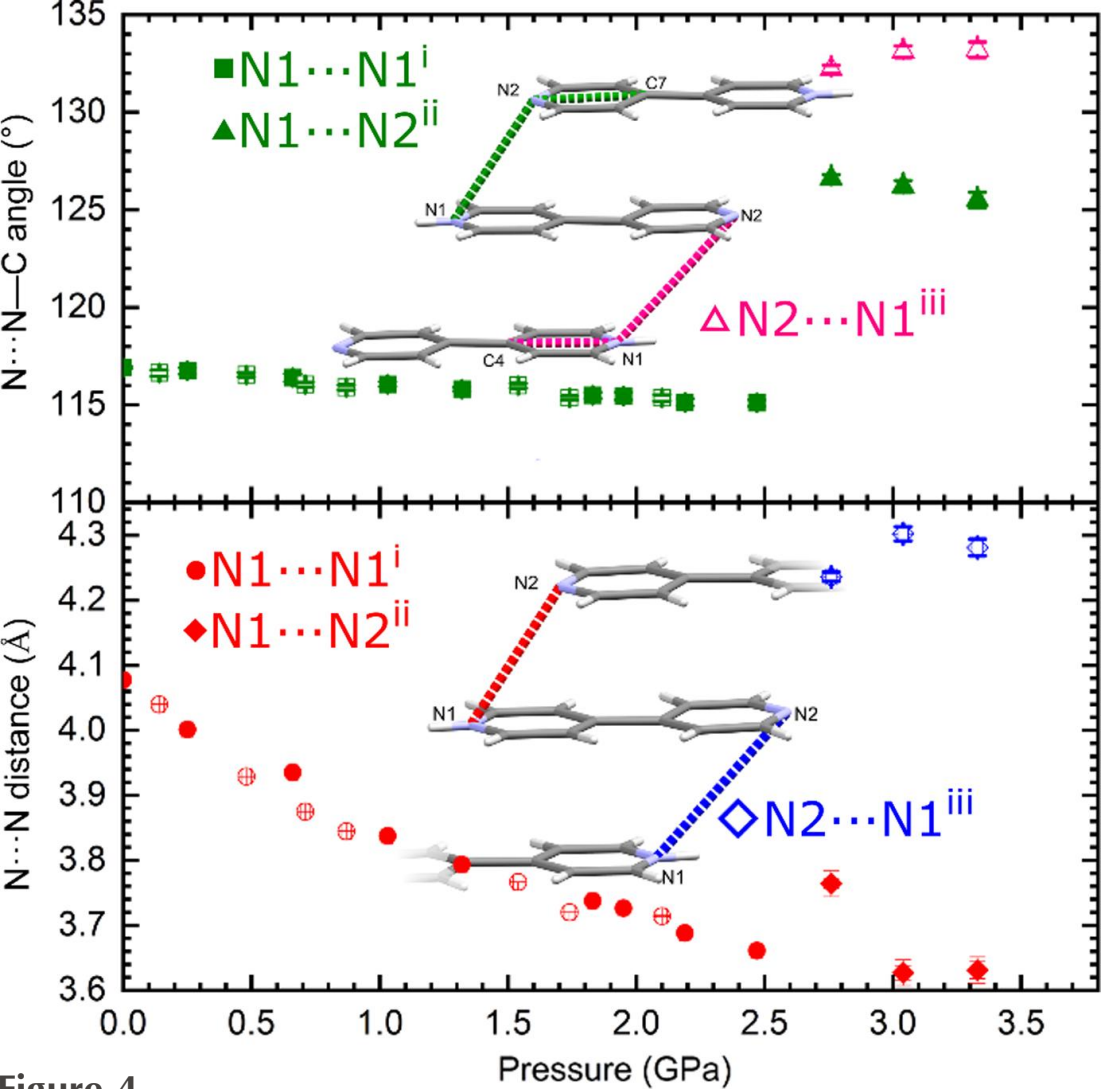
Pressure dependence of the N⋯N distances (bottom) and N⋯N⋯C angles (top) between stacked BIPY molecules/ions. Data for the *C*2/*c* and *P*2_1_/*c* phases are shown with circles/squares and diamond/triangles, respectively. Full symbols in the *C*2/*c* phase are for the compression series, and open for the decompression series. In the *P*2_1_/*c* phase, full symbols mark the data for the pyrimidine ring of BIPY with the N1 atom (shown in red and green), and open for the ring with the N2 atom (blue and pink). The way distances and angles were measured is presented in the figure inserted into the graph (for clarity, shown for the *P*2_1_/*c* phase only). Symmetry codes: (i) 1/2 − *x*, 1/2 − *y*, 1 − *z*; (ii) 1 − *x*, −*y*, 1 − *z*; (iii) 1 − *x*, 1 − *y*, 1 − *z*.

Experimentally and computationally, in the *C*2/*c* phase, the angle between stacked BIPY entities (*i.e.* the N1⋯N1⋯C4 angle, see Fig. 4 ▸) hardly changes on compression. XRD data show a decrease of less than 2°, from 116.9° at 0.1 MPa to 115.1° at 2.47 GPa. At the same time, the N1⋯N1 distance between two stacked BIPY entities decreases by over 10%, from 4.0772 (3) to 3.6609 (7) Å. As a result, the nitro­gen atoms become not only closer to each other but are also positioned more directly under one another. On transition to the *P*2_1_/*c* phase, at 3.33 GPa, the distance between nitro­gen atoms of neighbouring stacked 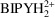 ions decreases slightly to 3.631 (13) Å for the N1⋯N2 (1 − *x*, −*y*, 1 − *z*) contact, and significantly increases to 4.281 (13) Å for the N2⋯N1 (1 − *x*, 1 − *y*, 1 − *z*) [two different values being the result of 1, not 0.5 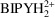 ion(s) being present in the asymmetric part of the unit cell]. At the same time the angle between stacked 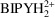 ions increases to 125.5 (4) and 133.2 (4)°, respectively. Therefore, even though one N⋯N distance becomes shorter, the nitro­gen atoms are not positioned as directly above one another as in the *C*2/*c* phase.

**Figure 5:**
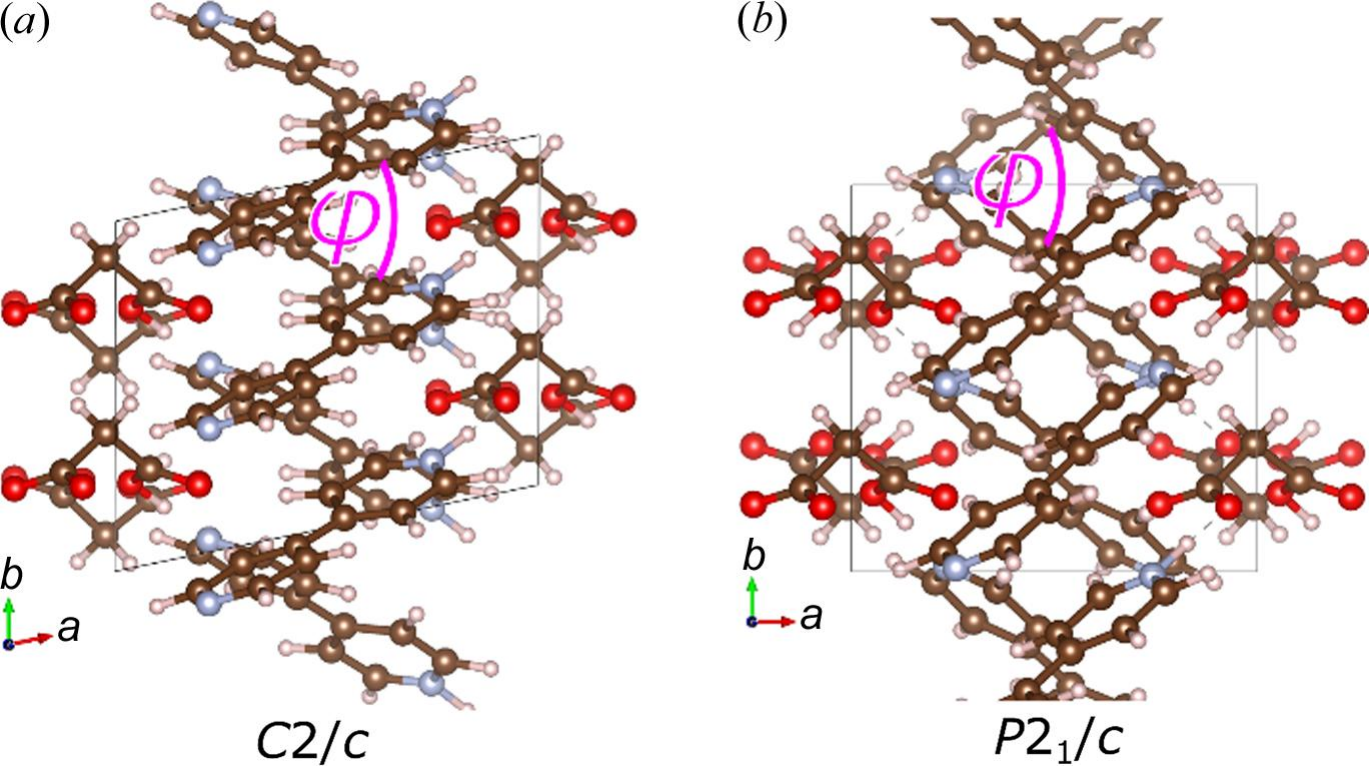
Structures of the (*a*) *C*2/*c* and (*b*) *P*2_1_/*c* phases represented in equivalent axes. The structure of the *C*2/*c* phase has been transformed and doubled along the *b* axis to have the same number of formula units as the *P*2_1_/*c* phase.

The shift of the BIPY entities and the resulting ripple effect are clearly visible for the structure shown along the [001] direction. In Fig. 5 ▸, the *C*2/*c* and the *P*2_1_/*c* structures projected along the *c* axis are presented. For the purpose of this comparison, the *C*2/*c* structure has been transformed and doubled along the *b* axis, resulting in comparable *b* and *c* axes between the two space groups. With this representation, the phase transition is associated with an increase of the γ angle from 78° in the *C*2/*c* phase to 90° in the *P*2_1_/*c* phase. The angle change causes 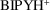 / 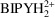 ions to open their relative angle ϕ in a scissor like movement. See Section S4 in the supporting information for further details.

Calculations of vibrational frequencies and Raman intensities at experimental volumes were performed after relaxing the atomic positions of the optimized *C*2/*c* and *P*2_1_/*c* phases at 1230 Å^3^ (0.1 MPa, neutral) and 1001 Å^3^ (3.1 GPa, diprotonated), respectively. In the case of the cocrystal, the agreement of experimental and calculated Raman spectra is good for both frequencies and relative intensities, particularly in the region from 1000 to 1600 cm^−1^. Although the agreement for the salt is not as satisfactory as for the cocrystal, the overall comparison is reasonable (see Fig. S7).

Our calculated Raman spectra exhibit features similar to those present in experimental spectra for neutral and diprotonated BIPY adsorbed in zeolites (Moissette *et al.*, 2001 ▸) despite these spectra being measured under ambient conditions. The most interesting feature is the splitting observed both in calculations and in experiments of the band at *ca* 1600 cm^−1^, characteristic for unprotonated BIPY. After double proton transfer, the splitting disappears and the main band moves closer to 1650 cm^−1^ (see Fig. S7). Unfortunately, insufficient resolution for the experimental Raman spectra does not allow for observation of the subtle band-splitting, still the shift towards higher values is visible. Overall, the computational results are consistent with the existence of the *C*2/*c* phase as neutral under ambient pressure and the *P*2_1_/*c* phase as a divalent salt at 3.1 GPa.

### 3.3. Single versus double proton transfer

As we established in the previous section, the symmetry change is associated with double proton transfer to the BIPY molecule. However, when the *P*2_1_/*c* phase was first observed at a pressure of 3.04 (2) GPa, the structure showed the length of the C—O bonds in one of the carb­oxy­lic groups becoming almost the same [1.25 (2) and 1.22 (2) Å], while the C—OH bond on the other side of the MA molecule elongated to 1.31 (2) Å (Fig. 3 ▸). This would suggest that only one proton was transferred to the BIPY molecule; however, this conclusion required confirmation with additional techniques as the bond lengths have significant estimated standard deviations. The experimental expectations were corroborated by DFT calculations, which established that the *P*2_1_/*c* phase is energetically favoured at volumes lower than 1150 Å^3^, with the range 1150–1000 Å^3^ being a metastable region where BIPY is monoprotonated (BIPYH^+^MA^−^), and below 1000 Å^3^ (*i.e.* at a pressure ≥3.1 GPa) where the 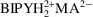 structure is stable. This outcome was independent from the symmetry of the space group, and the same results were yielded for the *C*2/*c* and *P*2_1_/*c* space groups.

Since the structure at 3.04 GPa, of unit-cell volume equal to 1001.5 (10) Å^3^, clearly shows discrepancies in the geometry of the carb­oxy­lic groups, consistent with the BIPYH^+^MA^−^ form, and at the same time is at the very limit of the metastability region, we postulate that the crystal was initially compressed to a volume slightly lower than 1000 Å^3^, triggering a double proton transfer, and then due to the relaxation of the DAC, the pressure dropped and transition from 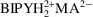 to BIPYH^+^MA^−^ occurred. This hypothesis can be corroborated by the data collected at 3.33 GPa, where we observe equalization of the carbon–oxygen bonds in both carb­oxy­lic groups of MA, regardless of their absolute length (with deviations from the 1.25 Å bond length for the delocalized carbon–oxygen bond being attributed to the quality of data affected by the use of the DAC). On decompression from 3.33 to 2.76 GPa, we again observe differences in geometry of the carb­oxy­lic groups of MA [1.295 (19) and 1.19 (2) Å versus 1.279 (19) and 1.240 (19) Å for C12—O1 and C12—O2 bonds and for C14—O3 and C14—O4 bonds, respectively]. This is in agreement with the result of theoretical calculations and previous observations at 3.04 GPa, with the structure of *P*2_1_/*c* symmetry containing monoprotonated BIPY, being preferred in the volume range 1000–1150 Å^3^. The variation in the carbon–oxygen bond length for *P*2_1_/*c* is presented in Fig. 3 ▸; however, note that the ‘middle’ point was measured for one crystal, and the remaining two pressure points for a different sample. In all three structures the atom labels and position of the molecule in the unit cell were selected in the same way to make the structural models uniform. For structures at 2.76 and 3.04 GPa, the symmetry is broken and only one carb­oxy­lic group is deprotonated (in each structure a different carb­oxy­lic group) and data points are shown with respect to a specific oxygen atom which may look like the bond length increases and decreases inconsistently, but the important feature is that the geometry is consistent with deprotonation for only one carb­oxy­lic group.

When compression of the crystal progresses from 0.1 MPa to 2.47 GPa, the O⋯N distance of the OH⋯N hydrogen bond connecting the MA and BIPY molecules into chains decreases (Figs. 3 ▸ and S10), which should favour proton transfer. The transition from BIPYMA to 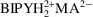 (at 3.33 GPa) results in further shortening of the O⋯N distances; however, on decompression to 3.04 and 2.76 GPa, when *P*2_1_/*c* symmetry is retained but the structure contains monoprotonated BIPY, they elongate, with some becoming even slightly longer than in *C*2/*c* phase at 2.47 GPa. Although the limitations coming from the quality of the structures cannot be discarded, the fact the O⋯N distances become longer can be a plausible explanation why there is a preference for monoprotonated salt at volumes higher than 1000 Å^3^ and why the proton transfer on one side of BIPY was reversed.

### 3.4. Putting pressure on the Δp*K*_a_ rule

To end the discussion of our results, we aim to explore whether a correlation between the Δp*K*_a_ rule and pressure-induced proton transfers in multicomponent crystals exists. Pressures observed by us for the first and second proton transfer in BIPYMA (0.2–1.3 and 3.1 GPa, respectively) point out that a larger increase in pressure is required to induce transfer of the second hydrogen atom, *i.e.* when Δp*K*_a_ is lower, since the corresponding Δp*K*_a_ values in these processes are 1.99 and −2.36, respectively.

**Figure 6:**
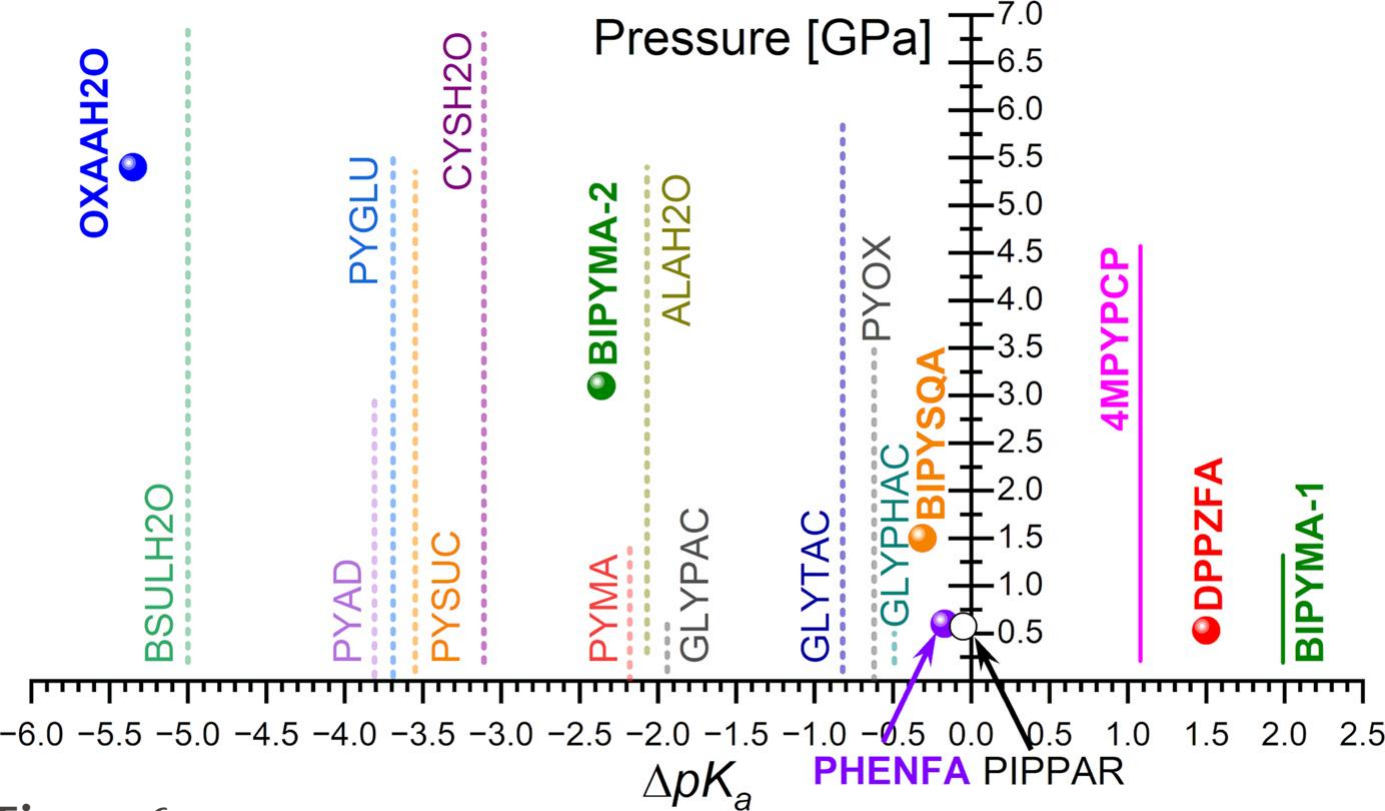
The proton-transfer pressure for confirmed reactions in multicomponent crystals as a function of Δp*K*_a_, marked with full circles and solid lines. Solid lines were used when not one specific pressure value was given (BIPYMA-1), or when the proton-transfer pressure was not reported (4MPYPCP). In the latter case, the whole investigated pressure range of the crystal was plotted. Open symbols and dotted lines (at 50% transparency) mark pressure ranges the selected multicomponent crystal structures deposited with the CSD were investigated in, and where the proton-transfer reaction was not observed. For details see Tables S6, S8 and S9. For PYAD, PYGLU, PYCUS, PYMA and PYOX, only Δp*K*_a_ for the first proton transfer was included as the Δp*K*_a_ for the second proton transfer is below −9 and falls outside the Δp*K*_a_ range considered in this work.

In this respect, the data reported so far are scarce, despite numerous multicomponent crystals being studied under pressure. To the best of our knowledge, identification of this process has only been accomplished in five systems: 4,4′-bipyridinium squarate – BIPYSQA (Reetz *et al.*, 1994 ▸; Martins *et al.*, 2009 ▸), oxalic acid dihydrate form α-OXAAH2O (Casati *et al.*, 2009 ▸), cocrystal of phenazine with fluoranilic acid – PHENFA (Kumai *et al.*, 2012 ▸), cocrystal of 2,3-di(2-pyridinyl)pyrazine with fluoranilic acid – DPPZFA (Horiuchi *et al.*, 2013 ▸), and cocrystal of 4-methylpyridine with pentachlorophenol – 4MPYPCP (Funnell *et al.*, 2021 ▸). Details of these experiments including Δp*K*_a_ and pressure values are provided in the supporting information and are collected in Table S6. The IR spectroscopic study of the quinhydrone crystal was excluded from the comparison as the reported proton-transfer reaction was cooperative with electron transfer (Mitani *et al.*, 1988 ▸). Fig. 6 ▸ summarizes the Δp*K*_a_ values and pressures at which the proton transfer occurs.

In principle, this small number of cases observed does not allow us to propose a conclusive trend. However, when our data and the previous results are all put together, our findings in the BIPYMA system can be generalized as a guideline in the following statement: the lower the Δp*K*_a_ value, the higher the pressure needed to initiate the proton-transfer process. For instance, in the range Δp*K*_a_ < 0, pressures promoting hydrogen transference decrease from around 5.4 GPa in OXAAH2O to 3.1 GPa in BIPYMA-2 and 1.5 GPa in the case of BIPYSQA. On the other hand, situations where the acid and the base are strong enough to show Δp*K*_a_ > 0, *i.e* the proton transference is favoured, pressures as low as 0.5 GPa or lower are sufficient to induce proton transfer. Thus, DPPZFA (Δp*K*_a_ = 1.5) shows a protonation pressure of 0.5 GPa, whereas the first proton transfer in BIPYMA (BIPYMA-1; showing Δp*K*_a_ > 1) starts at around 0.2 GPa and extends up to 1.3 GPa, (green vertical line in Fig. 6 ▸).

We believe that these results can be considered an extension of the Δp*K*_a_ rule. The capability of pressure to enhance interactions reducing interatomic distances between the acid and base functional groups of the coformers favours the protonation reaction. Shorter distances imply a higher affinity of the base for the acid hydrogen since a lower proton-migration barrier is expected, as has been discussed for instance in the case of hydrogen-bonding symmetrization (Meier *et al.*, 2022 ▸). This explains why the lower the Δp*K*_a_, the higher the pressure needed to initiate the proton-transfer process.

The implications of our findings can be revealed by considering other cases. For this purpose, the deposits of organic multicomponent crystals (excluding polymeric structures) investigated under high pressure were thoroughly analysed (see Table S7). Out of this subset of multicomponent crystals, seven systems: GLYPAC, CYSH2O, BSULH2O, ALAH2O, GLYTAC, PLYPHAC, PIPPAR [respective CSD refcode family names: AWIHOE (Zakharov *et al.*, 2013 ▸), CYSTAC (Johnstone *et al.*, 2009 ▸), IFIZIG (Johnstone *et al.*, 2009 ▸), IMEGIR (Zakharov & Boldyreva, 2013 ▸), IQOMIM (Losev *et al.*, 2016 ▸), NEPXIR (Losev *et al.*, 2016 ▸) and COKCEL (Oswald & Pulham, 2008 ▸)] were selected as potential candidates based on their chemical composition, a molecular arrangement enabling proton-transfer reaction, and Δp*K*_a_ values falling in the [−6,2] range (Table S8). However, protonation was not reported in either of these cases because it was not the main goal of the study or due to the experimental limitations.

According to our proposed extended Δp*K*_a_ rule, in BSULH2O, CYSH2O, ALAH2O and GLYTAC, the compression should have been sufficient to induce transfer of the hydrogen atom from the Brønsted–Lowry acid to the base. Interestingly, for the case of GLYPAC (Δp*K*_a_ = −1.94), GLYPHAC (Δp*K*_a_ = −0.49) and PIPPAR (Δp*K*_a_ = −0.05), we estimate, based on Fig. 6 ▸, that pressures between 2–3 GPa, 1–2 GPa and around 0.5–1 GPa, respectively, should produce the proton-transfer reaction.

In very recent work by Ward *et al.* (2023 ▸), where a series of pyridine–di­carb­oxy­lic acid systems was investigated under pressure using X-ray and neutron radiation, proton transfer was also not reported. However, in the context of the extended Δp*K*_a_ rule, and based on the Δp*K*_a_ values and the pressure limits for each of the studied cocrystals (Table S9) it could have been expected only in cocrystals of pyrazine with oxalic acid (PYOX, studied up to 3.5 GPa), succinic acid (PYSUC, studied up to 5.35 GPa) and glutaric acid (PYGLU, studied up to 5.5 GPa), and only for the single proton-transfer reaction. However, if those systems resemble BIPYMA, the first proton transfer might not have been clearly picked up by the diffraction techniques. The expected proton-transfer pressure for PYOX would be approximately 1.5 GPa, whereas for PYSUC and PYGLU pressure in the 4–4.5 GPa range should be sufficient to induce the first proton transfer. In fact, when carbon–oxygen bond lengths for PYSUC are analysed, a trend similar to that for BIPYMA is observed, where the single bond becomes shorter with pressure, while the double bond elongates (Fig. S15). For PYGLU and PYOX such clear trends were not visible (Figs. S16 and S17), but note that in the case of PYOX, most of the reported structures were of low resolution (0.9 Å). Nevertheless, drawing any conclusions on whether proton transfer took place or not based on the reported structural models alone would be biased and further studies with supporting techniques are required.

Although our results are encouraging, we cannot rule out the effect of the crystalline environment in the extended Δp*K*_a_ rule. Proton-transfer reactions anticipated under ambient conditions by this rule are not always observed, as explained by the recent results of Cruz-Cabeza *et al.* (2022 ▸). This turned out to be especially pertinent for the amino acids, as it was revealed that, for molecules existing in zwitterion form, the Δp*K*_a_ for a 50:50 salt:cocrystal ratio is set at 4.1, significantly higher than 1 observed for the whole population. Such an exceptional situation is explained as due to the particular spatial arrangement of the amino acids. This also suggests that the Δp*K*_a_ for preferential salt formation would be higher than expected as well. Therefore, higher pressures might be needed to trigger the reaction than those we suggest for the GLYPAC, GLYPHAC and PIPPAR multicomponent crystals. This would also explain why for BSULH2O, CYSH2O, ALAH2O and GLYTAC (all containing molecules of amino acids in the zwitterion form), proton transfer was not observed.

Unfortunately, the limited available data on pressure-induced proton-transfer reactions between different chemical entities in multicomponent crystals prevents establishing more precise rules about this phenomenon. Moreover, the current literature data hinder formulation of rules, based on the critical donor⋯acceptor distance at which the proton transfer takes place, similar to those expressed for polymerization reactions (Li *et al.*, 2021 ▸; Zhang, Tang *et al.*, 2022 ▸; Tang *et al.*, 2023 ▸). Out of the reported successful pressure-induced proton-transfer reactions in cocrystals, only two cases could be used for such analysis (BIPYMA and OXAAH2O). Other cases where proton transfer was reported were limited to single measurements, the crystal structure was not determined or the crystal structure did not show the proton transfer, making analysis impossible. In the case of BIPYMA, the donor⋯acceptor distance established by DFT calculations is approximately 2.55 Å, with the experimental values at 3.33 GPa falling below that limit [2.533 (14) and 2.529 (13) Å]. In OXAAH2O at 5.3 GPa, the donor⋯acceptor distance is 2.42 Å (structure OXACDH38) after proton transfer, compared with 2.43 Å at 3.6 GPa (structure OXACDH37) where molecules remain in neutral form. Note that for BIPYMA and OXAAH2O the chemical nature of the hydrogen bond involved in the proton transfer is different. In both cases the oxygen atom is a hydrogen atom donor, but the role of the acceptor is either taken by the nitro­gen atom of BIPY or the oxygen atom of the water molecule, which makes the two systems too different to establish a general donor⋯acceptor distance limit that would determine whether proton transfer takes place or not.

As the coformers in multicomponent crystals where the proton-transfer reaction was confirmed significantly differ in chemical structure and composition, it would be an oversight not to consider the pressure required for the reaction to take place being a result of several factors, including the crystal structure features, compressibility and chemical nature of the components. Although more research is needed, we believe that the extended Δp*K*_a_ rule we have shown in this study can serve as a guide.

## 4. Conclusions

Extensive high-pressure XRD and Raman spectroscopy experiments, coupled with the DFT calculations, have enabled identification of the relevant details of the expected intricate cocrystal–salt landscape of BIPYMA. Our results show that proton transfer to one of the pyridine rings of BIPY in the BIPYMA structure can occur already in the 0.2–1.3 GPa pressure range, with the mixture of BIPYMA and BIPYH^+^MA^−^ species present in the structure retaining the *C*2/*c* symmetry. BIPYMA and BIPYH^+^MA^−^ interconversion is possible due to the low-energy barrier (below 0.4 kcal mol^−1^ at a volume of 1150 Å^3^) and the low energy difference between both forms. On compression above 3.1 GPa, a double proton transfer occurs which triggers a phase transition. When nitro­gen atoms of BIPY get a formal positive charge, the accompanying repulsion can be released by spreading apart stacked 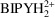 ions, resulting in lowering of the symmetry of the crystal to *P*2_1_/*c*. The *P*2_1_/*c* phase can be retained on decompression down to 2.4 GPa, with the structure containing monoprotonated BIPY (BIPYH^+^MA^−^) in this region, where the *P*2_1_/*c* phase is metastable. On further decompression to ambient conditions, the proton transfer (from monoprotonated to neutral BIPY) is reversed, similarly to a previously reported reaction observed in crystals of BIPYSQA.

Although, in light of this study, the proton transfer reaction in BIPYMA should not be considered as a continuous process from the perspective of the single O—H⋯N hydrogen bond, it can be described as such in regards to the crystal as a whole, meaning the transfer occurs entirely in some regions of the crystal and do not progress in others. The information we provide herein, in particular the metastability of the high-pressure *P*2_1_/*c* phase containing monoprotonated BIPY and the coexistence of BIPYMA and BIPYH^+^MA^−^ in the *C*2/*c* phase, shows the complexity of the response of multicomponent crystals amenable to proton transfer to high pressure and intricacies of the cocrystal–salt continuum.

Despite scattered examples of similar cases found in the literature, our comparison has shown a general trend where the value of the proton-transfer pressure is in an inverse relation to Δp*K*_a_. Further exploration of this topic can bring us closer to developing methods for targeted salt formation under high pressure. However, for this to happen it is crucial to be able to predict the required reaction-triggering pressure based on the structural information already available, without the need for cumbersome, time-consuming trial experiments for each individual acid–base pair. This highlights the need for a further thorough study of acid–base systems to fully understand the phenomenon of pressure-induced proton-transfer reactions in multicomponent crystals.

## 5. Related literature

The following references are cited in the supporting information: Abe *et al.* (2014 ▸); Andrzejewski *et al.* (2011 ▸, 2012 ▸); Anioła & Katrusiak (2016 ▸, 2017 ▸); Anioła *et al.* (2016 ▸); Bąkowicz & Turowska-Tyrk (2016 ▸, 2020 ▸, 2022 ▸); Bedeković *et al.* (2018 ▸); Bezzu *et al.* (2019 ▸); Bogdanov *et al.* (2020 ▸, 2022 ▸); Boschmann & Miller (2018 ▸); Budzianowski & Katrusiak (2006 ▸); Bujak & Angel (2006 ▸); Cameron *et al.* (2014 ▸); Carlsson *et al.* (2013 ▸); Chasák *et al.* (2021 ▸); Chia & Trimble (1961 ▸); Collings & Hanfland (2019 ▸); Connor *et al.* (2015 ▸); Craig *et al.* (2018 ▸); Dey & Lahiri (2010 ▸); Ehrenreich *et al.* (2019 ▸); Eikeland *et al.* (2016 ▸, 2017 ▸, 2020 ▸); Fabbiani *et al.* (2003 ▸, 2004 ▸, 2007 ▸, 2009 ▸, 2010 ▸, 2014 ▸, 2016 ▸); Filhol *et al.* (1981 ▸); Fornasari *et al.* (2020 ▸); Friedrich *et al.* (2020 ▸); Galica *et al.* (2020 ▸); Galica & Turowska-Tyrk (2019 ▸); Galiois *et al.* (1985 ▸, 1987 ▸, 1986 ▸); Galloway *et al.* (2010 ▸); Giordano *et al.* (2020 ▸); Granero–García *et al.* (2017 ▸); Guionneau *et al.* (1995 ▸, 2001 ▸); Guranda *et al.* (2012 ▸); Huq & Stephens (2006 ▸); Jaroń *et al.* (2020 ▸); Johnstone *et al.* (2008 ▸); Jungen *et al.* (2020 ▸); Katrusiak *et al.* (2011 ▸); Keller *et al.* (2018*a* ▸,*b* ▸); Khalili *et al.* (2009 ▸); Komatsu *et al.* (2020 ▸); Konieczny *et al.* (2015 ▸, 2016 ▸, 2017 ▸, 2018 ▸, 2020 ▸, 2021 ▸); Kortüm *et al.* (1960 ▸); Kozlenko *et al.* (2005 ▸); Kurnosov *et al.* (2004 ▸); LeBlanc *et al.* (2018 ▸); Le Pevelen *et al.* (1999 ▸); Lugo & Lubes (2007 ▸); Madsen *et al.* (2014 ▸); Mailman *et al.* (2017 ▸); Milašinović *et al.* (2021 ▸); Mínguez Espallargas *et al.* (2008 ▸); Moggach *et al.* (2009 ▸); Morency *et al.* (2021 ▸); Nam *et al.* (2009 ▸); Naumov *et al.* (2013 ▸); Nazeeruddin & Kalyanasundaram (1989 ▸); Nicholas *et al.* (2021 ▸); Nowicki *et al.* (2012 ▸); Olejniczak & Katrusiak (2010 ▸, 2011 ▸); Olejniczak *et al.* (2009 ▸, 2010 ▸, 2016 ▸, 2018 ▸, 2020 ▸, 2022*a* ▸,*b* ▸); Ono *et al.* (2018 ▸); Paliwoda *et al.* (2018 ▸); Parois *et al.* (2010 ▸); Patyk-Kaźmierczak & Kaźmierczak (2021 ▸); Patyk-Kaźmierczak *et al.* (2017 ▸); Pfrunder *et al.* (2020 ▸); Podsiadło *et al.* (2010 ▸); Poręba *et al.* (2019 ▸, 2020 ▸); Prescimone *et al.* (2009 ▸, 2010*a* ▸,*b* ▸); Price *et al.* (2022 ▸); Priola *et al.* (2022 ▸); Raghavendra (2022 ▸); Rejnhardt *et al.* (2021 ▸); Richardson *et al.* (2020 ▸); Rodríguez–Velamazán *et al.* (2014 ▸); Roszak & Katrusiak (2021 ▸); Saouane & Fabbiani (2015 ▸); Satthaphut *et al.* (2014 ▸); Schmitz *et al.* (2020 ▸); Schultz *et al.* (1986 ▸); Shepherd *et al.* (2012*a* ▸,*b* ▸, 2016 ▸); Shibaeva *et al.* (1985 ▸); Shimizu *et al.* (2000 ▸); Sobczak *et al.* (2020 ▸); Somayazulu *et al.* (1996 ▸); Starkey *et al.* (1986 ▸); Stevens *et al.* (2016 ▸); Szafrański (2014 ▸, 2020 ▸); Szafrański & Ståhl (2016 ▸); Terlecki *et al.* (2021 ▸); Thiel *et al.* (2020 ▸); Wallenfels & Friedrich (1960 ▸); Ward *et al.* (2023 ▸); Ward & Oswald (2019 ▸); Woodall *et al.* (2016 ▸); Wu *et al.* (2015 ▸); Yamaura & Kato (2002 ▸); Zakharov *et al.* (2015 ▸); Zhang *et al.* (2022 ▸); Zieliński & Katrusiak (2015 ▸, 2016 ▸).

## Supplementary Material

Crystal structure: contains datablock(s) BIPYMA, BIPYMA_014, BIPYMA_025, BIPYMA_048, BIPYMA_066, BIPYMA_071, BIPYMA_087, BIPYMA_103, BIPYMA_132, BIPYMA_154, BIPYMA_174, BIPYMA_183, BIPYMA_195, BIPYMA_210, BIPYMA_219, BIPYMA_247, BIPYMA_276, BIPYMA_304_rc, BIPYMA_333. DOI: 10.1107/S2052252524000344/yc5045sup1.cif

Structure factors: contains datablock(s) BIPYMA. DOI: 10.1107/S2052252524000344/yc5045BIPYMAsup2.hkl

Structure factors: contains datablock(s) BIPYMA_014. DOI: 10.1107/S2052252524000344/yc5045BIPYMA_014sup3.hkl

Structure factors: contains datablock(s) BIPYMA_025. DOI: 10.1107/S2052252524000344/yc5045BIPYMA_025sup4.hkl

Structure factors: contains datablock(s) BIPYMA_048. DOI: 10.1107/S2052252524000344/yc5045BIPYMA_048sup5.hkl

Structure factors: contains datablock(s) BIPYMA_066. DOI: 10.1107/S2052252524000344/yc5045BIPYMA_066sup6.hkl

Structure factors: contains datablock(s) BIPYMA_071. DOI: 10.1107/S2052252524000344/yc5045BIPYMA_071sup7.hkl

Structure factors: contains datablock(s) BIPYMA_087. DOI: 10.1107/S2052252524000344/yc5045BIPYMA_087sup8.hkl

Structure factors: contains datablock(s) BIPYMA_103. DOI: 10.1107/S2052252524000344/yc5045BIPYMA_103sup9.hkl

Structure factors: contains datablock(s) BIPYMA_132. DOI: 10.1107/S2052252524000344/yc5045BIPYMA_132sup10.hkl

Structure factors: contains datablock(s) BIPYMA_154. DOI: 10.1107/S2052252524000344/yc5045BIPYMA_154sup11.hkl

Structure factors: contains datablock(s) BIPYMA_174. DOI: 10.1107/S2052252524000344/yc5045BIPYMA_174sup12.hkl

Structure factors: contains datablock(s) BIPYMA_183. DOI: 10.1107/S2052252524000344/yc5045BIPYMA_183sup13.hkl

Structure factors: contains datablock(s) BIPYMA_195. DOI: 10.1107/S2052252524000344/yc5045BIPYMA_195sup14.hkl

Structure factors: contains datablock(s) BIPYMA_210. DOI: 10.1107/S2052252524000344/yc5045BIPYMA_210sup15.hkl

Structure factors: contains datablock(s) BIPYMA_219. DOI: 10.1107/S2052252524000344/yc5045BIPYMA_219sup16.hkl

Structure factors: contains datablock(s) BIPYMA_247. DOI: 10.1107/S2052252524000344/yc5045BIPYMA_247sup17.hkl

Structure factors: contains datablock(s) BIPYMA_276. DOI: 10.1107/S2052252524000344/yc5045BIPYMA_276sup18.hkl

Structure factors: contains datablock(s) BIPYMA_304_rc. DOI: 10.1107/S2052252524000344/yc5045BIPYMA_304_rcsup19.hkl

Structure factors: contains datablock(s) BIPYMA_333. DOI: 10.1107/S2052252524000344/yc5045BIPYMA_333sup20.hkl

Movie S1: pressure-induced changes in the crystal morphology. DOI: 10.1107/S2052252524000344/yc5045sup21.gif

Movie S1: pressure-induced changes in the crystal morphology. DOI: 10.1107/S2052252524000344/yc5045sup22.mp4

Supporting figures and tables. DOI: 10.1107/S2052252524000344/yc5045sup23.pdf

CCDC references: 2279875, 2279876, 2279877, 2279878, 2279879, 2279880, 2279881, 2279882, 2279883, 2279884, 2279885, 2279886, 2279887, 2279888, 2279889, 2279890, 2279891, 2279892, 2279893
