# Supplementary figures and images for: The curious case of proton migration under pressure in the malonic acid and 4,4′-bi­pyridine cocrystal
Source: IUCrJ. 2024 Jan 13;11(Pt 2):168–81. doi: 10.1107/S2052252524000344 (PMC10916288; doi:10.1107/S2052252524000344)

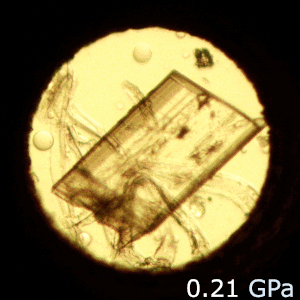

Supplement: Supplementary file 21 [file m-11-00168-sup21.gif]
