# Supplementary material for: The curious case of proton migration under pressure in the malonic acid and 4,4′-bi­pyridine cocrystal
Source: IUCrJ. 2024 Jan 13;11(Pt 2):168–81. doi: 10.1107/S2052252524000344 (PMC10916288; doi:10.1107/S2052252524000344)
Supplement: Supplementary file 23 [file m-11-00168-sup23.pdf]

IUCrJ

Volume 11 (2024)

Supporting information for article:

The curious case of proton migration under pressure in the malonic acid and 4,4'-bipyridine cocrystal

Ewa Patyk-Kaźmierczak, Fernando Izquierdo-Ruiz, Alvaro Lobato, Michał Kaźmierczak, Ida Moszczyńska, Anna Olejniczak and J. Manuel Recio

S1. Additional experimental details

S1.1. Acidic hydrogen atom refinement

For structures up to 0.4 GPa, the hydrogen was localized at MA molecule. For structures in 0.4-1.4 GPa range, structures were refined with hydrogen atom disordered between MA and BIPY if the difference in the length of the C=O and C-O bonds was lower than 0.04 Å (otherwise the H-atom was localized at MA). All structures in *C*2/*c* phase in 1.4-2.5 GPa range, were refined with hydrogen atom disordered between MA and BIPY. In $P2_1/c$ phase the hydrogen atom was assigned to BIPY if the difference in the length of the C=O and C-O bonds of the H-bonded carboxylic group of MA molecule was lower than 0.04 Å, and if the difference was higher it was localized at MA.

S1.2. DFT calculations

To analyse the stability and the proton transfer of the *C*2/*c* and the $P2_1/c$ phases, three independent optimisations at each volume, for both the *C*2/*c* and the $P2_1/c$ space groups, were performed: starting with (i) the protons at the malonic acid, (ii) one proton transferred to the bipyridine and, (iii) two protons transferred. As a result, in total, 6 different starting guesses have been used to explore each volume point, three for the cocrystal space group and three for the salt space group.

The proton transfer transition barrier was computed in the *C*2/*c* space group at fixed volume of 1150 $Å^3$. The O-H distances were fixed at different values and an optimization of the rest of the cell was performed. We applied the same computational parameters and convergence criteria as described previously for the optimizations.

We would like to notice that the accuracy of the computational strategy carried out in our study can be improved following more time-consuming procedures as discussed by LeBlanc *et al.* (2018) This alternative has been shown to be prohibitive in our calculations and does not meaningfully affect the trends found and the comparison with the experimental results.

S2. Tables

S2.1. XRD experimental tables

Table S1. Experimental details

	(BIPYMA)	(BIPYMA_014)	(BIPYMA_025)	(BIPYMA_048)
Crystal data				
Chemical formula	$C_3H_4O_4 \cdot C_{10}H_8N_2$	$C_3H_4O_4 \cdot C_{10}H_8N_2$	$C_3H_4O_4 \cdot C_{10}H_8N_2$	$C_3H_4O_4 \cdot C_{10}H_8N_2$
Form	BIPYMA	BIPYMA	BIPYMA	BIPYMA
Type of experiment	ambient	HP decompression	HP compression	HP decompression
M_r	260.25	260.25	260.25	260.25
Crystal system	Monoclinic	Monoclinic	Monoclinic	Monoclinic,
Space group	$C2/c$	$C2/c$	$C2/c$	$C2/c$
p (GPa)	0.0001	0.14(2)	0.25(2)	0.48(2)
T (K)	298	298	298	298
a, b, c (Å)	19.5688(9), 4.0772(3), 15.9523(8)	19.550(5), 4.0396(3), 15.854(5)	19.561(8), 4.0010(5), 15.776(9)	19.582(7), 3.9289(4), 15.625(6)
β (°)	104.783(5)	104.96(4)	105.08(6)	105.30(5)
V (Å^3)	1230.64(13)	1209.6(5)	1192.2(9)	1159.5(7)
Z/Z'	4/0.5	4/0.5	4/0.5	4/0.5
Radiation type	Cu $K\alpha$	Mo $K\alpha$	Mo $K\alpha$	Mo $K\alpha$
μ (mm^{-1})	0.89	0.11	0.11	0.11
Crystal size (mm)		0.23 × 0.14 × 0.06	0.35 × 0.20 × 0.02	0.28 × 0.12 × 0.02
Data collection				
Diffractometer	SuperNova, Atlas	New Xcalibur, EosS2	Xcalibur, Eos	New Xcalibur, EosS2
Absorption correction	Analytical[a]	Gaussian[b]	Gaussian[b]	Gaussian[b]
T_{min}, T_{max}	0.874, 0.956	0.455, 0.577	0.453, 0.577	0.452, 0.577
No. of measured, independent and observed reflections	6263, 1256, 834 [$I \geq 2u(I)$]	6263, 804, 306 [$I > 2\sigma(I)$]	4852, 784, 253 [$I > 2\sigma(I)$]	5802, 755, 316 [$I > 2\sigma(I)$]
R_{int}	0.039	0.121	0.154	0.103
$(\sin \theta/\lambda)_{max}$ (Å^{-1})	0.630	0.670	0.663	0.675
Refinement				
$R[F^2 > 2\sigma(F^2)]$, $wR(F^2)$, S	0.037, 0.111, 1.09	0.080, 0.225, 1.00	0.066, 0.149, 1.03	0.061, 0.174, 1.01
No. of reflections	1256	804	784	755
No. of parameters	141	88	88	88
No. of restraints	0	18	60	19
H-atom treatment	All H-atom parameters refined	H-atom parameters constrained	H-atom parameters constrained	H-atom parameters constrained
$\Delta\rho_{max}$, $\Delta\rho_{min}$ (e Å^{-3})	0.23, −0.28	0.17, −0.16	0.17, −0.16	0.15, −0.13

Table S1. Experimental details- *continuation*.

	(BIPYMA_066)	(BIPYMA_071)	(BIPYMA_087)	(BIPYMA_103)
Crystal data				
Chemical formula	$C_3H_4O_4{\cdot}C_{10}H_8N_2$	$C_3H_4O_4{\cdot}C_{10}H_8N_2$	$C_3H_4O_4{\cdot}C_{10}H_8N_2$	$C_3H_4O_4{\cdot}C_{10}H_8N_2$
Form	BIPYMA	BIPYMA	BIPYMA	BIPYMA
Type of experiment	HP compression	HP decompression	HP decompression	HP compression
M_r	260.25	260.25	260.25	260.25
Crystal system	Monoclinic	Monoclinic	Monoclinic	Monoclinic,
Space group	*C*2/*c*	*C*2/*c*	*C*2/*c*	*C*2/*c*
p (GPa)	0.66(2)	0.71(2)	0.87(2)	1.03(2)
T (K)	298	298	298	298
a, *b*, *c* (Å)	19.581(6), 3.9351(5), 15.640(7)	19.604(5), 3.8747(3), 15.531(4)	19.612(5), 3.8444(3), 15.462(4)	19.598(9), 3.8374(5), 15.442(9)
β (°)	105.31(4)	105.37(3)	105.61(3)	105.76(7)
V (Å^3)	1162.4(7)	1137.5(4)	1122.8(4)	1117.7(9)
Z/*Z'*	4/0.5	4/0.5	4/0.5	4/0.5
Radiation type	Mo *K*α	Mo *K*α	Mo *K*α	Mo *K*α
μ (mm^{-1})	0.11	0.12	0.12	0.12
Crystal size (mm)	0.35 × 0.15 × 0.01	0.28 × 0.12 × 0.02	0.28 × 0.12 × 0.02	0.33 × 0.15 × 0.03
Data collection				
Diffractometer	Xcalibur, Eos	New Xcalibur, EosS2	New Xcalibur, EosS2	Xcalibur, Eos
Absorption correction	Gaussian[b]	Gaussian[b]	Gaussian[b]	Gaussian[b]
T_{min}, T_{max}	0.454, 0.577	0.451, 0.577	0.448, 0.577	0.449, 0.576
No. of measured, independent and observed reflections	2535, 721, 195 [$I > 2\sigma(I)$]	5895, 738, 336 [$I > 2\sigma(I)$]	5440, 730, 323 [$I > 2\sigma(I)$]	5693, 714, 298 [$I > 2\sigma(I)$]
R_{int}	0.136	0.096	0.101	0.134
$(\sin \theta/\lambda)_{max}$ (Å^{-1})	0.672	0.670	0.674	0.665
Refinement				
$R[F^2 > 2\sigma(F^2)]$, $wR(F^2)$, S	0.089, 0.275, 0.97	0.055, 0.150, 1.08	0.064, 0.177, 1.08	0.074, 0.135, 1.03
No. of reflections	721	738	730	714
No. of parameters	88	88	88	88
No. of restraints	42	42	36	12
H-atom treatment	H-atom parameters constrained	H-atom parameters constrained	H-atom parameters constrained	H-atom parameters constrained
$\Delta\rho_{max}$, $\Delta\rho_{min}$ (e Å^{-3})	0.21, −0.20	0.21, −0.19	0.22, −0.18	0.20, −0.16

Table S1. Experimental details- *continuation*.

	(BIPYMA_132)	(BIPYMA_154)	(BIPYMA_174)	(BIPYMA_183)
Crystal data				
Chemical formula	$C_{10}H_{8.353}N_2·C_3H_{3.647}O_4$	$C_3H_{3.559}O_4·C_{10}H_{8.441}N_2$	$C_3H_{3.402}O_4·C_{10}H_{8.598}N_2$	$C_3H_{3.414}O_4·C_{10}H_{8.586}N_2$
Form	BIPYMA/BIPYH$^+$MA$^-$	BIPYMA/BIPYH$^+$MA$^-$	BIPYMA/BIPYH$^+$MA$^-$	BIPYMA/BIPYH$^+$MA$^-$
Type of experiment	HP compression	HP decompression	HP decompression	HP compression
M_r	260.25	260.25	260.25	260.25
Crystal system	Monoclinic	Monoclinic	Monoclinic	Monoclinic,
Space group	*C*2/*c*	*C*2/*c*	*C*2/*c*	*C*2/*c*
p (GPa)	1.32(2)	1.54(2)	1.74(2)	1.83(2)
T (K)	298	298	298	298
a, *b*, *c* (Å)	19.631(5), 3.7932(3), 15.352(4)	19.635(3), 3.7664(2), 15.296(3)	19.647(4), 3.7198(3), 15.191(4)	19.647(6), 3.7375(4), 15.222(6)
β (°)	105.95(3)	106.03(2)	106.21(3)	106.12(4)
V (Å^3)	1099.2(4)	1087.2(3)	1066.1(4)	1073.8(6)
Z	4	4	4	4
Radiation type	Mo *K*α	Mo *K*α	Mo *K*α	Mo *K*α
μ (mm^{-1})	0.12	0.12	0.12	0.12
Crystal size (mm)	0.33 × 0.15 × 0.03	0.28 × 0.12 × 0.02	0.28 × 0.12 × 0.02	0.33 × 0.15 × 0.03
Data collection				
Diffractometer	Xcalibur, Eos	New Xcalibur, EosS2	New Xcalibur, EosS2	Xcalibur, Eos
Absorption correction	Gaussian[b]	Gaussian[b]	Gaussian[b]	Gaussian[b]
T_{min}, T_{max}	0.450, 0.576	0.452, 0.577	0.452, 0.577	0.449, 0.575
No. of measured, independent and observed [$I > 2\sigma(I)$] reflections	5808, 718, 289	5433, 711, 349	5209, 690, 329	3130, 633, 247
R_{int}	0.130	0.100	0.108	0.129
$(\sin \theta/\lambda)_{max}$ (Å^{-1})	0.667	0.674	0.675	0.667
Refinement				
$R[F^2 > 2\sigma(F^2)]$, $wR(F^2)$, S	0.062, 0.138, 1.05	0.063, 0.113, 1.06	0.070, 0.180, 1.03	0.067, 0.154, 1.05
No. of reflections	718	711	690	633
No. of parameters	89	89	89	89
No. of restraints	32	43	43	47
H-atom treatment	H-atom parameters constrained	H-atom parameters constrained	H-atom parameters constrained	H-atom parameters constrained
$\Delta\rho_{max}$, $\Delta\rho_{min}$ (e Å^{-3})	0.18, −0.17	0.25, −0.19	0.20, −0.19	0.20, −0.24

Table S1. Experimental details- *continuation*.

	(BIPYMA_195)	(BIPYMA_210)	(BIPYMA_219)	(BIPYMA_247)
Crystal data				
Chemical formula	$C_3H_{3.599}O_4·C_{10}H_{8.401}N_2$	$C_3H_{3.392}O_4·C_{10}H_{8.608}N_2$	$C_3H_{3.209}O_4·C_{10}H_{8.791}N_2$	$C_3H_{3.406}O_4·C_{10}H_{8.594}N_2$
Form	BIPYMA/BIPYH$^+$MA$^-$	BIPYMA/BIPYH$^+$MA$^-$	BIPYMA/BIPYH$^+$MA$^-$	BIPYMA/BIPYH$^+$MA$^-$
Type of experiment	HP compression	HP decompression	HP compression	HP compression
M_r	260.25	260.25	260.25	260.25
Crystal system	Monoclinic	Monoclinic	Monoclinic	Monoclinic,
Space group	$C2/c$	$C2/c$	$C2/c$	$C2/c$
p (GPa)	1.95(2)	2.10(2)	2.19(2)	2.47(2)
T (K)	298	298	298	298
M_r	260.25	260.25	260.25	260.25
a, *b*, *c* (Å)	19.662(9), 3.7264(6), 15.211(10)	19.648(17), 3.7139(11), 15.171(17)	19.670(5), 3.6881(3), 15.112(5)	19.687(13), 3.6609(7), 15.043(13)
β (°)	106.16(7)	106.08(12)	106.48(4)	106.48(9)
V (Å^3)	1070.4(10)	1063.7(17)	1051.3(5)	1039.6(12)
Z	4	4	4	4
Radiation type	Mo *K*α	Mo *K*α	Mo *K*α	Mo *K*α
μ (mm^{-1})	0.12	0.12	0.12	0.13
Crystal size (mm)	0.33 × 0.15 × 0.03	0.26 × 0.11 × 0.02	0.35 × 0.20 × 0.02	0.35 × 0.20 × 0.02
Data collection				
Diffractometer	Xcalibur, Eos	Xcalibur, Eos	Xcalibur, Eos	Xcalibur, Eos
Absorption correction	Gaussian[b]	Gaussian[b]	Gaussian[b]	Gaussian[b]
T_{min}, T_{max}	0.451, 0.576	0.451, 0.576	0.450, 0.577	0.456, 0.576
No. of measured, independent and observed [$I > 2σ(I)$] reflections	2630, 585, 226	4521, 684, 258	2411, 623, 231	2467, 615, 217
R_{int}	0.139	0.139	0.129	0.137
(sin θ/λ)$_{max}$ (Å^{-1})	0.668	0.658	0.668	0.669
Refinement				
$R[F^2 > 2σ(F^2)]$, $wR(F^2)$, S	0.077, 0.194, 1.03	0.079, 0.236, 0.97	0.093, 0.215, 1.10	0.074, 0.111, 1.01
No. of reflections	585	684	623	615
No. of parameters	89	89	89	89
No. of restraints	44	43	25	43
H-atom treatment	H-atom parameters constrained	H-atom parameters constrained	H-atom parameters constrained	H-atom parameters constrained
$Δρ_{max}$, $Δρ_{min}$ (e Å^{-3})	0.25, −0.21	0.26, −0.25	0.22, −0.22	0.18, −0.24

Table S1. Experimental details- *continuation*.

	(BIPYMA_276)	(BIPYMA_304_rc)	(BIPYMA_333)
Crystal data			
Chemical formula	$C_3H_3O_4·C_{10}H_9N_2$	$C_3H_3O_4·C_{10}H_9N_2$	$C_3H_2O_4·C_{10}H_{10}N_2$
Form	$BIPYH^+MA^-$	$BIPYH^+MA^-$	$BIPYH_2^{2+}MA^{2-}$
Type of experiment	HP decompression	HP compression	HP compression
M_r	260.25	260.25	260.25
Crystal system	Monoclinic	Monoclinic	Monoclinic
Space group	$P2_1/c$	$P2_1/c$	$P2_1/c$
p (GPa)	2.76(2)	3.04(2)	3.33(2)
T (K)	298	298	298
a, *b*, *c* (Å)	8.412(4), 7.9665(11), 15.576(9)	8.438(6), 7.9032(14), 15.425(10)	8.40(1), 7.886(3), 15.389(19)
β (°)	103.33(6)	103.18(8)	103.93(14)
V (Å^3)	1015.7(8)	1001.5(10)	989.5(18)
Z	4	4	4
Radiation type	Mo *K*α	Mo *K*α	Mo *K*α
μ (mm^{-1})	0.13	0.13	0.13
Crystal size (mm)	0.34 × 0.17 × 0.08	0.28 × 0.12 × 0.02	0.37 × 0.19 × 0.09
Data collection			
Diffractometer	Xcalibur, Eos	New Xcalibur, EosS2	New Xcalibur, EosS2
Absorption correction	Analytical[a]	Gaussian[b]	Gaussian[b]
T_{min}, T_{max}	0.987, 0.990	0.452, 0.578	0.636, 0.732
No. of measured, independent and observed [$I > 2\sigma(I)$] reflections	4177, 998, 409	2932, 947, 269	2861, 888, 335
R_{int}	0.082	0.145	0.099
$(\sin θ/λ)_{max}$ (Å^{-1})	0.664	0.663	0.665
Refinement			
$R[F^2 > 2\sigma(F^2)]$, $wR(F^2)$, S	0.062, 0.162, 1.02	0.064, 0.162, 0.91	0.095, 0.216, 1.02
No. of reflections	998	947	888
No. of parameters	173	173	172
No. of restraints	83	91	86
H-atom treatment	H-atom parameters constrained	H-atom parameters constrained	H-atom parameters constrained
$\Delta\rho_{max}$, $\Delta\rho_{min}$ (e Å^{-3})	0.19, −0.21	0.21, −0.20	0.28, −0.25

[a]*CrysAlis PRO* 1.171.41.93a (Rigaku Oxford Diffraction, 2020) Analytical numeric absorption correction using a multifaceted crystal model based on expressions derived by R.C. Clark & J.S. Reid. (Clark, R. C. & Reid, J. S. (1995). Acta Cryst. A51, 887-897) Empirical absorption correction using spherical harmonics, implemented in SCALE3 ABSPACK scaling algorithm

[b] *CrysAlis PRO* 1.171.41.93a (Rigaku Oxford Diffraction, 2020) Numerical absorption correction based on gaussian integration over a multifaceted crystal model (Absorb Angel (2004) J. Appl. Cryst. 37:486-492)

Table 1 Table S2. Unit-cell parameters and order of the short experiments aimed at establishing the pressure limits for *C*2/*c* and $P2_1/c$ phases. Each section of the table (separated by a thick black line) represent data for an individual sample crystal that was measured. Additionally, information about the phase is included (*C*2/*c* highlighted in purple and $P2_1/c$ in orange). The ordinal numbers (O.N.) signify the order in which the crystal was measured. For all crystals the compression is considered a ‘rapid’ compression, as X-ray diffraction measurements were short (approx. 3h) and sample did not remain under given pressure for longer than several hours.

O.N.	Phase	*p* (GPa)	*a* (Å)	*b* (Å)	*c* (Å)	*β* (°)	*V* (Å³)	Experiment info
1st	$P2_1/c$	3.09(2)	8.43(3)	7.902(3)	15.47(7)	103.4(5)	1003(6)	Compression
2nd	$C2/c$	2.37(2)	19.7(2)	3.705(3)	15.1(2)	107(2)	1052(20)	Decompression
1st	$P2_1/c$	3.12(2)	8.44(5)	7.849(10)	15.37(4)	102.1(4)	996(6)	Compression
2nd	$P2_1/c$	3.03(2)	8.409(13)	7.883(4)	15.424(12)	103.25(23)	995(2)	Compression
3rd	$P2_1/c$	3.60(2)	8.44(3)	7.812(5)	15.21(2)	103.2(3)	977(4)	Compression
4th	$P2_1/c$	2.88(2)	8.485(19)	7.878(5)	15.473(18)	102.94(19)	1008(3)	Decompression
5th	$P2_1/c$	2.81(2)	8.44(2)	7.955(8)	15.518(16)	103.65(18)	1012(3)	Decompression
6th	$P2_1/c$	2.70(2)	8.416(11)	7.972(3)	15.593(10)	103.65(10)	1017(2)	Decompression
7th	$P2_1/c$	2.52(2)	8.34(2)	8.007(5)	15.604(15)	103.70(17)	1013(3)	Decompression
8th	$C2/c$	1.14(2)	19.91(8)	3.775(5)	15.32(4)	105.5(4)	1109(6)	Decompression
9th	$P2_1/c$	2.86(2)	8.51(4)	7.973(14)	15.34(3)	102.7(3)	1015(6)	Compression
10th	$C2/c$	2.11(2)	19.9(2)	3.691(7)	15.18(17)	106(1)	1072(17)	Decompression (further compression of the sample led to its destruction and poor diffracting properties)
1st	$C2/c$	2.86(2)	19.89(16)	3.628(3)	14.93(3)	106.8(5)	1032(8)	Compression
2nd	$C2/c$	2.39(2)	19.6(2)	3.680(4)	15.15(4)	106.8(6)	1044(11)	Decompression from 3.37 GPa
3rd	$P2_1/c$	2.40(2)	8.50(3)	8.025(4)	15.648(2)	103.10(17)	1039(4)	Decompression from 3.35 GPa
4th	$C2/c$	2.95(2)	20.0(2)	3.563(6)	14.81(17)	104(1)	1021(15)	Compression

Table S3. The order of high-pressure experiments used for solving and refinement of the crystal structures. Each colour section of the table represent data for an individual sample crystal that was measured. Additionally, information about the phase is included. The ordinal numbers (O.N.) signify the order in which the crystal was measured. The 'slow' compression mode refers to samples that were compressed gradually from ambient pressure (or decompressed). The gradual nature was achieved by performing approx. 24h long measurement at each pressure stage, letting sample to rest under specific pressure for at least 1 day. For 'rapid' compression mode the pressure was achieved directly without leaving sample crystal under lower pressures for a long period of time.

O.N.	Phase	*p* (GPa)	Name of the experiment	Compression mode
1st	$C2/c$	0.25(2)	BIPYMA_025	Rapid
2nd	$C2/c$	0.66(2)	BIPYMA_066	Slow
3rd	$C2/c$	1.03(2)	BIPYMA_103	Slow
4th	$C2/c$	1.32(2)	BIPYMA_132	Slow
5th	$C2/c$	1.83(2)	BIPYMA_183	Slow
6th	$C2/c$	1.95(2)	BIPYMA_195	Slow
7th	$C2/c$	2.19(2)	BIPYMA_219	Slow
8th	$C2/c$	2.47(2)	BIPYMA_247	Slow
9th	$C2/c$	2.10(2)	BIPYMA_210	Slow
10th	$C2/c$	1.74(2)	BIPYMA_174	Slow
11th	$C2/c$	1.54(2)	BIPYMA_154	Slow
12th	$C2/c$	0.87(2)	BIPYMA_087	Slow
13th	$C2/c$	0.71(2)	BIPYMA_071	Slow
14th	$C2/c$	0.48(2)	BIPYMA_048	Slow
15th	$C2/c$	0.14(2)	BIPYMA_014	Slow
16th	$P2_1/c$	3.04(2)	BIPYMA_304_rc	Rapid
1st	$P2_1/c$	3.33(2)	BIPYMA_333	Rapid
2nd	$P2_1/c$	2.76(2)	BIPYMA_276	Slow

S2.2. Tables summarizing DFT results

Table S4. DFT results for the $C2/c$ space group. Protons header describes the initial and final protonation states, e.g. 0P→1P means that the initial guess structure is the neutral form and the final optimized structure contains one transferred proton from MA to BIPY. E/u.f. is energy per unit formula relative to the lowest energy of all computed structures.

V (Å^3)	a (Å)	b (Å)	c (Å)	β (°)	E/u.f. (kJ·mol^{-1})	Protons
1300.0	19.84	4.06	16.61	103.6	5.9	0P→0P
1300.0	19.70	4.06	16.75	104.1	5.0	1P→0P
1300.0	19.52	4.06	17.01	105.2	4.6	2P→0P
1250.0	19.87	3.98	16.29	103.8	2.1	0P→0P
1250.0	19.56	4.02	16.45	104.6	2.9	1P→1P
1250.0	19.60	3.95	16.74	105.2	1.7	2P→0P
1230.6	19.63	3.90	16.63	104.9	1.3	0P→0P
1200.0	19.89	3.89	15.98	104.0	0.8	0P→0P
1200.0	19.64	3.92	16.14	104.9	0.0	1P→1P
1200.0	19.71	3.84	16.42	105.3	0.8	2P→0P
1150.0	19.92	3.81	15.67	104.5	2.1	0P→0P
1150.0	19.72	3.82	15.83	105.2	0.0	1P→1P
1150.0	19.55	3.81	16.09	106.1	2.5	2P→2P
1100.0	19.90	3.71	15.44	105.1	7.9	0P→0P
1100.0	19.78	3.71	15.56	105.7	4.6	1P→1P
1100.0	19.64	3.70	15.75	106.2	5.4	2P→2P
1050.0	19.75	3.63	15.22	106.1	13.0	0P→2P
1050.0	19.86	3.61	15.26	106.3	15.1	1P→1P
1050.0	19.72	3.61	15.39	106.5	13.4	2P→2P
1000.0	19.81	3.52	14.97	106.9	29.7	0P→2P
1000.0	19.79	3.52	15.04	107.2	29.3	1P→2P
1000.0	19.83	3.49	15.11	107.2	30.1	2P→2P
978.2	19.88	3.47	14.86	107.3	40.6	0P→2P
950.0	19.88	3.42	14.66	107.7	58.2	0P→2P
950.0	19.81	3.42	14.76	108.2	57.3	1P→2P
950.0	19.92	3.39	14.78	108.0	58.6	2P→2P

Table S5. DFT results for the $P2_1/c$ space group. Protons header describes the initial and final protonation states, e.g. 0P→1P means that the initial guess structure is the neutral form and the final optimized structure contains one transferred proton from MA to BIPY. E/u.f. is energy per unit formula relative to the lowest energy of all computed structures.

V (Å^3)	a (Å)	b (Å)	c (Å)	β (°)	E/u.f. (kJ·mol^{-1})	Protons
1300.0	9.43	8.36	16.78	100.9	7.5	0P→0P
1300.0	9.57	8.29	16.68	101.0	7.5	1P→0P
1300.0	9.26	8.51	16.79	100.6	7.1	2P→0P
1250.0	9.02	8.43	16.75	101.0	3.8	0P→0P
1250.0	9.11	8.48	16.51	101.3	4.6	1P→1P
1250.0	8.91	8.58	16.61	100.3	3.8	2P→0P
1200.0	8.89	8.30	16.60	101.5	1.7	0P→0P
1200.0	8.96	8.33	16.43	101.8	1.3	1P→1P
1200.0	8.80	8.48	16.40	101.4	1.3	2P→1P
1150.0	8.78	8.16	16.41	101.9	2.5	0P→0P
1150.0	8.80	8.20	16.32	102.4	0.4	1P→1P
1150.0	8.67	8.32	16.30	102.2	0.0	2P→1P
1100.0	8.66	8.06	16.20	103.0	2.5	0P→1P
1100.0	8.68	8.06	16.15	102.9	2.5	1P→1P
1100.0	8.49	8.28	16.05	102.7	2.5	2P→2P
1050.0	8.63	7.87	15.89	103.2	10.5	0P→1P
1050.0	8.58	7.90	15.91	103.4	10.0	1P→1P
1050.0	8.39	8.10	15.89	103.5	7.1	2P→2P
1000.0	8.52	7.77	15.57	103.8	20.9	0P→2P
1000.0	8.48	7.79	15.61	104.1	20.5	1P→2P
1000.0	8.40	7.84	15.66	104.2	20.5	2P→2P
1001.6	8.38	7.90	15.57	103.6	19.7	1P→2P
971.0	8.38	7.76	15.35	103.7	33.9	1P→2P
950.0	8.54	7.55	15.18	103.9	47.7	0P→2P
950.0	8.77	7.30	15.40	105.5	49.4	1P→2P
950.0	8.47	7.58	15.27	104.3	47.3	2P→2P

S2.3. Tables summarizing literature and CSD analysis

Table S6. List of multicomponent crystals for which pressure-induced proton transfer reaction was reported in the literature and for BIPYMA.

Compound acronym	Acid	pK_a		Base	pK_a		ΔpK_a	p_{PT}		Acid-Base Reaction
		Value	Ref.		Value	Ref.		Value [GPa]	Ref.	
DPPZFA	fluoranilic acid	1.4	(Wallenfels & Friedrich, 1960)	2,3-di(2-pyridinyl)pyrazine	2.9	(Nazeeruddin & Kalyanasundaram, 1989)	1.5	0.53	(Horiuchi *et al.*, 2013)	$C_6H_2F_2O_4+C_{14}H_{10}N_4 \rightarrow C_6HF_2O_4^-+C_{14}H_{11}N_4^+$
PHENFA	fluoranilic acid	1.4	(Wallenfels & Friedrich, 1960)	phenazine	1.23	(Bedeković *et al.*, 2018)	-0.17	0.6	(Kumai *et al.*, 2012)	$C_6H_2F_2O_4+C_{12}H_8N_2 \rightarrow C_6HF_2O_4^-+C_{12}H_9N_2^+$
OXAAH2O-x[a]	oxalic acid	3.61	(Lugo & Lubes, 2007)	water	-1.74	(Starkey *et al.*, 1986)	-5.35	2	(Bhatt *et al.*, 2016)	$C_2H_2O_4+2H_2O \rightarrow C_2O_4^{2-}+2H_3O^+$
OXAAH2O[a]	oxalic acid	3.61	(Lugo & Lubes, 2007)	water	-1.74	(Starkey *et al.*, 1986)	-5.35	5.4	(Casati *et al.*, 2009)	$C_2H_2O_4+2H_2O \rightarrow C_2O_4^{2-}+2H_3O^+$
4MPYPCP	pentachlorophenol	4.9	(Morency *et al.*, 2021)	4-methylpyridine	5.98	(Shimizu *et al.*, 2000)	1.08	0.21-4.57[b]	(Funnell *et al.*, 2021)	$C_6HCl_5O+C_6H_7N \rightarrow C_6Cl_5O^-+C_6H_8N^+$
BIPYSQA	squarate anion	3.58	(Chasák *et al.*, 2021)	monoprotonated 4,4'-bipyridinium cation	3.27	(Ulstrup *et al.*, 1969)	-0.31	1.5	(Ma *et al.*, 2017)	$C_4H_1O_4^-+C_{10}H_9N_2^+ \rightarrow C_4O_4^{2-}+C_{10}H_{10}N_2^{2+}$
BIPYMA	malonic acid	2.83	(Khalil *et al.*, 2013)	4,4'-bipyridine	4.82	(Ulstrup *et al.*, 1969)	1.99	0.19-1.32	This work	$C_3H_4O_4 + C_{10}H_8N_2 \rightarrow C_3H_3O_4^-+C_{10}H_9N_2^+$
BIPYMA	hydrogen malonate anion	5.63	(Khalil *et al.*, 2013)	monoprotonated 4,4'-bipyridinium cation	3.27	(Ulstrup *et al.*, 1969)	-2.36	3.1	This work	$C_3H_3O_4^-+C_{10}H_9N_2^+ \rightarrow C_3H_2O_4^{2-}+C_{10}H_{10}N_2^{2+}$

[a] In case of oxalic acid dihydrate form α two values of pressure of proton transfer can be found in the literature. Original study by Casati et al.(2009) from 2009 reported formation of hydronium ion above 5.3 GPa (OXAAH2O). The reaction was hypothesized based on the changes in the geometry of carboxylic group of the acid and DFT calculations, and was later confirmed by neutron diffraction and spectroscopic studies.(Macchi *et al.*, 2010) However, subsequent IR study by Bhatt et al.(2016) reported that the proton migration in oxalic acid dihydrate might actually start at much lower pressure of 2 GPa (OXAH2O-x), with pure ionic phase archived at approx. 5 GPa. Therefore, in the discussion in the main manuscript the value of 5.3 GPa was discussed as it corresponds to complete transformation from neutral to ionic phase. Interestingly, a study of form β of oxalic acid dihydrate did not lead to proton-transfer reaction, despite both polymorphs exhibiting quite similar geometries of the O–H···O hydrogen bonds at ambient conditions (with O···O distances equal 2.51 and 2.52 Å in form α and β, respectively) (Macchi *et al.*, 2010). The different pressure response of polymorph β was explained by the less favourable orientation of the water molecule.

[b] The exact pressure for proton-transfer reaction (p_{PT}) was not given by the authors; therefore the whole pressure range sample was investigated in is given.

Table S7. List of REFCODE families of multicomponent crystals (excluding polymers) studied under high-pressure and deposited in the CSD. Salts are highlighted in pink, cocrystals in green, clathrates in grey, hydrates in blue, solvates in orange and cocrystals of salts in purple. Two refcode families for structures for which proton transfer was reported are listed in red.

REFCODE family	Pressure range (GPa)	N° of HP deposits	Type of the crystal	Ref.
AHADUL	1.00-4.00	2	solvated salt	(Pfrunder *et al.*, 2020)
AJOBAC	0.34	1	hydrate	(Wu *et al.*, 2015)
AKEYOG	0.84-2.17	4	solvate	(Zielinski & Katrusiak, 2016)
AKEZIB	0.92-2.31	6	solvate	(Zielinski & Katrusiak, 2016)
AWIHOE	0.10-0.70	2	cocrystal	(Zakharov *et al.*, 2013)
AXEZUA	1.05	1	cocrystal	(Anioła *et al.*, 2016)
AZOCNI	0.58-1.65	4	solvated salt	(Craig *et al.*, 2018)
BALWEQ	0.50	1	solvated salt	(Le Pevelen *et al.*, 1999)
BAMZUN	0.38-8.40	8	cocrystal	(Eikeland *et al.*, 2016)
BANBOK	3.60	1	cocrystal	(Eikeland *et al.*, 2016)
BANFII	4.80	1	cocrystal	(Eikeland *et al.*, 2016)
BEWWIK	0.80	1	clathrate	(Kurnosov *et al.*, 2004)
BIHXIC	0.40-12.68	11	salt	(Paliwoda *et al.*, 2018)
BIJBUU	10.66	1	salt	(Paliwoda *et al.*, 2018)
BILNOC	14.70-15.19	2	salt	(G. Ehrenreich *et al.*, 2019)
BIZJUP	0.33-2.06	3	salt	(Poręba *et al.*, 2020)
BOBVIY	0.20-1.00	4	salt	(Olejniczak *et al.*, 2010)
BOLDIP	0.38-1.73	5	salt	(Szafrański, 2014)
BPYRDB	0.10-1.00	4	salt	(Anioła & Katrusiak, 2016)
CAQQAP	1.00	1	salt	(Konieczny *et al.*, 2017)
CARGIM	0.30-0.90	2	salt	(Konieczny *et al.*, 2015)
CECKOO	0.10-1.60	4	salt	(Priola *et al.*, 2022)
CIJMUG	3.20	1	cocrystal	(Ono *et al.*, 2018)
CIJNAN	3.80	1	cocrystal	(Ono *et al.*, 2018)
CILHIO	0.15-0.46	2	salt	(Schultz *et al.*, 1986)
COKCEL	0.57	1	solvated cocrystal	(H. Oswald & R. Pulham, 2008)
COTPHP	0.54-9.20	10	salt	(Thiel *et al.*, 2020)
CUJQUW	2.70-8.00	9	clathrate	(Eikeland *et al.*, 2020)
CUJWEM	0.54-3.80	5	clathrate	(Eikeland *et al.*, 2020)
CYSTAC	0.20-6.80	6	hydrate	(Johnstone *et al.*, 2009)
DATREV	0.95	1	salt	(Shibaeva *et al.*, 1985)

Table S7. *Continuation.*

REFCODE family	Pressure range (GPa)	Nº of HP deposits	Type of the crystal	Ref.
DICWAN	0.11-0.60	3	salt	(Szafrański & Ståhl, 2016)
DUSBEY	0.55-1.10	3	salt	(Guionneau *et al.*, 1995)
ECUQOI	0.30	1	cocrystal	(Olejniczak *et al.*, 2009)
ECUTUR	0.17-22.56	24	cocrystal	(Friedrich *et al.*, 2020)
EGEVOE	2.68-3.43	3	cocrystal	(Collings & Hanfland, 2019)
EGEWEV	3.83-8.82	10	cocrystal	(Collings & Hanfland, 2019)
EGEYEX	9.51-26.19	14	cocrystal	(Collings & Hanfland, 2019)
EKESAQ	4.25-6.90	4	clathrate	(Eikeland *et al.*, 2016)
EKESEU	4.30	1	clathrate	(Eikeland *et al.*, 2016)
EKESIY	0.18-6.20	10	cocrystal	(Eikeland *et al.*, 2016)
ETACOU	0.25-1.51	4	salt	(Nicholas *et al.*, 2021)
EVEDAN	0.75	1	salt	(Konieczny *et al.*, 2021)
EVEDER	0.75	1	salt	(Konieczny *et al.*, 2021)
EVUQAO	0.50-1.40	2	hydrated salt	(Olejniczak & Katrusiak, 2011)
FOXNUB	0.29-1.87	8	salt	(Szafrański, 2014)
GADGUN*	0.21-4.57	7	cocrystal	(Funnell *et al.*, 2021)
GISKOI	0.87-4.09	9	solvate	(Price *et al.*, 2022)
GUSHAD	0.14-4.09	21	hydrate	(Patyk-Kaźmierczak & Kaźmierczak, 2021)
HALTAP	0.60-12.80	8	salt	(Poręba *et al.*, 2019)
HOJYAI	0.30-0.90	6	salt	(Konieczny *et al.*, 2015)
HQUACN	0.30-14.10	17	clathrate	(Eikeland *et al.*, 2017)
HUSXUO	0.80-7.50	12	clathrate	(Eikeland *et al.*, 2020)
IFIZIG	0.20-6.90	8	hydrate	(Johnstone *et al.*, 2009)
IKIVUW*	0.21	1	cocrystal	(Funnell *et al.*, 2021)
IMEGIR	0.30-5.40	9	hydrated salt	(Zakharov & Boldyreva, 2013)
IQOMIM	0.10-5.91	12	cocrystal	(Losev *et al.*, 2016)
IZIYIC	0.13-3.96	12	salt	(Szafrański, 2020)
IZIYOI	0.13-2.39	15	hydrated salt	(Szafrański, 2020)
JAFBUO	0.14-1.89	8	salt	(Shepherd *et al.*, 2016)
KADWIX	0.13-0.70	3	hydrate	(Wu *et al.*, 2015)
KARJOG	0.12-1.27	15	salt	(Rejnhardt *et al.*, 2021)
KEKZAB	0.99-4.97	4	solvate	(Schmitz *et al.*, 2020)
KELLOG	0.10-0.55	5	hydrate	(Olejniczak *et al.*, 2022*a*)
KESYIQ	0.25-1.20	3	salt	(Prescimone, Sanchez-Benitez, Kamenev, Moggach *et al.*, 2010)
KICCOO	0.10-7.80	10	hydrate	(Johnstone *et al.*, 2009)

Table S7. *Continuation.*

REFCODE family	Pressure range (GPa)	Nº of HP deposits	Type of the crystal	Ref.
KIWXIZ	0.86-3.00	4	cocrystal	(Stevens *et al.*, 2016)
KODVAD	0.38-2.67	6	cocrystal	(Bezzu *et al.*, 2019)
LAJLAL	0.16-1.76	11	solvated salt	(Patyk-Kaźmierczak *et al.*, 2017)
LETGIA	0.36-1.51	4	salt	(Katrusiak *et al.*, 2011)
LIFNOE	0.60-0.60	1	hydrate	(Fabbiani *et al.*, 2007)
LILKOJ	1.06	1	hydrated salt	(Olejniczak *et al.*, 2018)
LIZFIK	0.25-1.60	5	solvate	(Shepherd, Rosa *et al.*, 2012)
LOCCAI	0.12-0.60	6	hydrated salt	(Olejniczak *et al.*, 2016)
LSERMH	1.00-5.80	7	hydrate	(Johnstone *et al.*, 2008)
LUDQIN	3.30	1	cocrystal	(Komatsu *et al.*, 2020)
LUKWAS	0.40-1.59	4	cocrystal	(Sobczak *et al.*, 2020)
LUKWOG	1.95-2.50	2	cocrystal	(Sobczak *et al.*, 2020)
MAWMIJ	0.30-1.15	6	salt	(Anioła & Katrusiak, 2017)
MENTIJ	1.00-4.00	4	salt	(Bujak & Angel, 2006)
MESEFV	0.15-0.98	5	salt	(Galiois *et al.*, 1985), (Gallois *et al.*, 1987), (Gallois *et al.*, 1986)
MEWXOE	0.16-4.50	5	salt	(Keller, Prescimone, Bolink *et al.*, 2018)
MEWZIA	3.50	1	salt	(Keller, Prescimone, Bolink *et al.*, 2018)
MOFFUJ	0.30-1.00	5	cocrystal	(Bąkowicz & Turowska-Tyrk, 2020)
MUJPEP	0.10-1.49	6	cocrystal of salt	(Fornasari *et al.*, 2020)
MUWMUP	0.50-1.50	2	salt	(Jungen *et al.*, 2020)
NEDMIS	1.14-5.98	6	salt	(Connor *et al.*, 2015)
NEPXIR	0.19-0.50	2	cocrystal	(Losev *et al.*, 2016)
NPOFNP	0.28-18.92	17	cocrystal	(Friedrich *et al.*, 2020)
NUFZUM	4.70-8.10	10	cocrystal	(Giordano *et al.*, 2020)
NUPVUQ	0.55	1	solvated salt	(Anioła & Katrusiak, 2017)
NURKOB	1.50	1	solvate	(Parois *et al.*, 2010)
NUYFIX	0.45-4.55	5	solvated salt	(Prescimone, Sanchez-Benitez, Kamenev, Warren *et al.*, 2010)
NUYGOE	0.18-2.00	3	solvated salt	(Prescimone, Sanchez-Benitez, Kamenev, Warren *et al.*, 2010)
OHAVUQ	0.54-1.49	4	hydrate	(Zieliński & Katrusiak, 2015)
OHAXIF	0.87-1.84	2	salt	(Moggach *et al.*, 2009)
OHUGOO	2.50	1	salt	(Prescimone, Sanchez-Benitez, Kamenev, Moggach *et al.*, 2010)
OHUJUX	0.21-0.90	2	salt	(Prescimone, Sanchez-Benitez, Kamenev, Moggach *et al.*, 2010)
OMISIM	0.62-0.75	3	solvate	(Fabbiani *et al.*, 2003), (Ward & Oswald, 2019)
ONUROG	0.21-0.41	2	salt	(Olejniczak *et al.*, 2016)
ONUTOI	0.21-2.38	4	salt	(Olejniczak *et al.*, 2016)

Table S7. *Continuation.*

REFCODE family	Pressure range (GPa)	Nº of HP deposits	Type of the crystal	Ref.
OTELEH	0.10-6.52	16	salt	(Bogdanov *et al.*, 2022)
OXACDH	1.60-5.30	3	hydrate/salt	(Casati *et al.*, 2009)
OXUDOD	4.80-9.70	6	cocrystal	(Jaroń *et al.*, 2020)
PAQUBR	0.35	1	salt	(Anioła & Katrusiak, 2016)
PARZUG	0.30-4.80	7	salt	(Naumov *et al.*, 2013)
PASBIX	2.20	1	salt	(Naumov *et al.*, 2013)
PASXIS	0.48-1.50	3	hydrated salt	(Andrzejewski *et al.*, 2011)
PAXBAV	0.10-0.40	3	cocrystal	(Bąkowicz & Turowska-Tyrk, 2022)
PAXBID	0.10	1	cocrystal	(Bąkowicz & Turowska-Tyrk, 2022)
PESBAT	0.25-6.00	18	salt	(Bogdanov *et al.*, 2020), (Milašinović *et al.*, 2021)
PIGQUS	0.30-2.20	3	hydrated salt	(Galloway *et al.*, 2010)
POWSID	0.20-2.48	6	solvate	(Shepherd, Palamarciuc *et al.*, 2012)
PYRDHN	1.10	1	salt	(Kozlenko *et al.*, 2005)
QEJPAX	1.00	1	salt	(Huq & Stephens, 2006)
QIYYEF	0.50-2.00	5	cocrystal	(Galica & Turowska-Tyrk, 2019)
QOVKEU	0.20-0.80	2	salt	(Abe *et al.*, 2014)
QUTQIH	0.15-6.85	12	salt	(Zakharov *et al.*, 2015)
RASBEV	0.76	1	hydrate	(Granero-García *et al.*, 2017)
RASBIZ	0.61	1	hydrate	(Granero-García *et al.*, 2017)
RAYZEZ	1.17-6.20	6	clathrate	(Eikeland *et al.*, 2017)
RAZBAY	6.70-8.60	4	clathrate	(Eikeland *et al.*, 2017)
REQCIC	0.30-3.30	5	salt	(Keller, Prescimone, Constable *et al.*, 2018)
REQCUO	1.00-4.50	5	salt	(Keller, Prescimone, Constable *et al.*, 2018)
REYFUW	0.55	1	salt	(Bujak & Angel, 2006)
RIBKES	0.32-3.20	7	solvate	(Madsen *et al.*, 2014)
SANFUM	0.10-0.90	7	hydrate	(Olejniczak *et al.*, 2022*b*)
SAZZID	0.35-4.10	8	salt	(Mínguez Espallargas *et al.*, 2008)
SEHHIX	0.45-3.73	8	salt	(Mínguez Espallargas *et al.*, 2008)
SOFPEJ	0.10-1.00	3	salt	(Konieczny *et al.*, 2021)
SUVGEW	1.80	1	solvated salt	(Olejniczak & Katrusiak, 2010)
SUVGIA	1.00-2.40	4	solvated salt	(Olejniczak & Katrusiak, 2010), (Andrzejewski *et al.*, 2012)
TABREX	0.10-0.58	2	hydrate	(Olejniczak *et al.*, 2020)
TABRIB	0.23	1	hydrate	(Olejniczak *et al.*, 2020)
TAVKUX	0.40-1.80	4	cocrystal of salt	(Carlsson *et al.*, 2013)
TAWTAM	1.05-6.55	6	salt	(Wong *et al.*, 2013)

Table S7. *Continuation.*

REFCODE family	Pressure range (GPa)	Nº of HP deposits	Type of the crystal	Ref.
TIQDUT	0.10-4.30	6	solvate	(Woodall *et al.*, 2016)
TMAMPT	0.25-1.92	7	salt	(Richardson *et al.*, 2020)
TTFTCQ	0.46	1	salt	(Filhol *et al.*, 1981)
TUCZIB	0.16-1.25	3	salt	(Prescimone *et al.*, 2009)
TUVROU	0.20	1	salt	(Konieczny *et al.*, 2020)
TUVXAL	0.50-4.80	6	salt	(Satthaphut *et al.*, 2014)
UBAPIZ	0.50-1.00	6	cocrystal	(Bąkowicz & Turowska-Tyrk, 2016)
UBAQIA	0.50-1.20	2	cocrystal	(Bąkowicz & Turowska-Tyrk, 2016)
UBAQOG	0.50	1	cocrystal	(Bąkowicz & Turowska-Tyrk, 2016)
UHITUB	0.25	1	hydrated cocrystal of salt	(Fabbiani *et al.*, 2009)
UHUTOH	0.62	1	cocrystal	(Olejniczak *et al.*, 2009)
UTUSIO	0.52-1.10	3	hydrate	(Roszak & Katrusiak, 2021)
VABWON	0.50	1	solvate	(Fabbiani *et al.*, 2016)
VASZAT	4.40-6.10	2	solvate	(Mailman *et al.*, 2017)
VASZIB	2.00	1	solvate	(Mailman *et al.*, 2017)
WAFNAT	1.10	1	hydrate	(Fabbiani *et al.*, 2004)
WAMJAW	0.70-1.35	2	cocrystal of salt	(Yamaura & Kato, 2002)
WATDUR	0.10-1.00	6	hydrate	(Olejniczak *et al.*, 2022*a*)
WIFNAB	1.80	1	salt	(Konieczny *et al.*, 2016)
WOLYUR	0.41-1.20	4	salt	(Olejniczak *et al.*, 2018)
WOLZAY	0.30-0.78	3	salt	(Nowicki *et al.*, 2012), (Budzianowski & Katrusiak, 2006)
XAJMUS	1.80	1	solvate	(Podsiadło *et al.*, 2010)
XECMIB	0.16-0.40	2	solvate	(Guionneau *et al.*, 2001)
XEFPEF	2.30	1	salt	(Rodríguez-Velamazán *et al.*, 2014)
XIKSAO	1.30	1	salt	(Konieczny *et al.*, 2018)
XOLQUN	0.10-3.88	11	cocrystal	(Bezzu *et al.*, 2019)
XOYFUP	0.50	2	cocrystal	(Galica *et al.*, 2020)
XOYGIE	0.50	1	cocrystal	(Galica *et al.*, 2020)
XUVJAB	0.22	1	hydrate	(Wu *et al.*, 2015)
XUZHIM	2.84	1	cocrystal	(Friedrich *et al.*, 2020)
YACZOV	0.12-2.61	8	solvate	(Terlecki *et al.*, 2021)
YAWYED	3.30	1	cocrystal	(Ono *et al.*, 2018)
YIVGIV	0.11	1	salt	(Olejniczak *et al.*, 2016)
YUJNIC	0.20	1	hydrated salt	(Saouane & Fabbiani, 2015)
YUJPIE	0.27	1	hydrated salt	(Saouane & Fabbiani, 2015)

Table S7. *Continuation.*

REFCODE family	Pressure range (GPa)	N^o of HP deposits	Type of the crystal	Ref.
YUJPOK	0.60-1.14	2	hydrated salt	(Saouane & Fabbiani, 2015)
YUXZIB	0.13-1.93	3	salt	(Cameron *et al.*, 2014)
YUZTET	0.90	1	hydrate	(Fabbiani *et al.*, 2010)
ZADMID	0.14	1	solvate	(Stevens *et al.*, 2016)
ZAKNIL	1.00	1	salt	(Konieczny *et al.*, 2018)
ZEFJAX	1.20-1.70	2	solvated salt	(Andrzejewski *et al.*, 2012)
ZIPWOM	0.44	1	hydrate	(Fabbiani *et al.*, 2014)
ZOSXOU	6.20	1	cocrystal	(Somayazulu *et al.*, 1996)
ZOSXOW	7.90	1	cocrystal	(Somayazulu *et al.*, 1996)
ZOSXUA	5.60	1	cocrystal	(Somayazulu *et al.*, 1996)
ZZZVLI	0.80-3.10	5	solvate	(Eikeland *et al.*, 2017)

Table S8. List of selected multicomponent crystals investigated under high pressure, with crystal structures reported in the CSD, that contain acid-base pairs capable of undergoing proton-transfer reaction, but such reaction was not observed and with ΔpK_a in -6 to 2 range. Crystals are named with the names of REFCODE families they belong to. Pressure range the crystals were investigated in (p) are also listed.

REFCODE family (acronym)	Name	Acid	pK_a		Base	pK_a		ΔpK_a	p (GPa)
			Value	ref		Value	ref		
AWIHOE (GLYPAC)	ammonioacetate pentanedioic acid	pentanedioic acid	4.34	(Boschmann & Miller, 2018)	glycine	2.4	(Guranda *et al.*, 2012)	-1.94	0.10-0.70
COKCEL (PIPAR)	paracetamol hemikis(piperazine) ethanol solvate	paracetamol	9.78	(Nam *et al.*, 2009)	piperazine	9.73	(Khalili *et al.*, 2009)	-0.05	0.57
CYSTAC (CYSH2O)	2-ammonio-2-carboxyethanesulfonate monohydrate	L-cysteic acid (carboxylic group)	1.37	[a]	water	-1.74	(Starkey *et al.*, 1986)	-3.11	0.20-6.80
IFIZIG (BSULH2O)	4-(2-ammonio-2-carboxyethyl)benzenesulfonate monohydrate	4-(2-ammonio-2-carboxyethyl) benzenesulfonate	3.26	[a]	water	-1.74	(Starkey *et al.*, 1986)	-5.0	0.20-6.90
IMEGIR (ALAH2O)	DL-alaninium semi-oxalate monohydrate	DL-alaninium	2.33	(Dey & Lahiri, 2010)	water	0.26	(Starkey *et al.*, 1986)	-2.07	0.30-5.40
IQOMIM (GLYTAC)	glycine DL-tartaric acid	DL-tartaric acid	3.22	(Kortüm *et al.*)	glycine	2.4	(Guranda *et al.*, 2012)	-0.82	0.10-5.91
NEPXIR (GLYPHAC)	glycine phthalic acid	phthalic acid	2.89	(Raghavendra, 2022)	glycine	2.4	(Guranda *et al.*, 2012)	-0.49	0.19-0.50

[a] When reliable literature source for experimental pK_a value could not be found the value was calculated using Chemicalize from ChemAxon (https://chemicalize.com/, date of calculations September 6th 2023).(Chemicalize)

Table S9. List of cocrystals of pyrazine with dicarboxylic acids studied under high-pressure conditions by Ward *et al* (2023), including the pK_a and ΔpK_a values for the coformers, and the pressure range the cocrystals were studied in.

REFCODE family (acronym)	Name	Acid	pK_a		Base	pK_a		ΔpK_a	*p* (GPa)
			Value	ref		Value	ref		
GUDSUV (PYOX)	pyrazine oxalic acid	oxalic acid	1.27	(Khalil *et al.*, 2013)	pyrazine	0.65	(Chia & Trimble, 1961)	-0.62	0.0001-3.48
			3.51			-5,78		-9.39	
VAXWAU (PYSUC)	pyrazine succinic acid	succinic acid	4.19	(Khalil *et al.*, 2013)	pyrazine	0.65	(Chia & Trimble, 1961)	-3.54	0.1-5.35
			5.65			-5,78		-11.43	
GUDTOQ (PYGLU)	pyrazine glutaric acid	glutaric acid	4.34	(Boschmann & Miller, 2018)	pyrazine	0.65	(Chia & Trimble, 1961)	-3.69	0.05-5.5
			5.41			-5,78		-11.19	
GUDVAE (PYAD)	pyrazine adipic acid	adipic acid	4.46	(Khalil *et al.*, 2013)	pyrazine	0.65	(Chia & Trimble, 1961)	-3.81	0.05-3.0
			5.43			-5,78		-11.21	
GUDTEG* (PYMA)	pyrazine bis(malonic acid)/pyrazine malonic acid	malonic acid	2.83	(Khalil *et al.*, 2013)	pyrazine	0.65	(Chia & Trimble, 1961)	-2.18	0.0001-1.48
			5.63			-5,78		-11.41	

*Other associated REFCODE family names: LOBHET, LOBHIX, COBHOD, LOBHUJ, LOBJAR, LOBJEV

S3. Figures

S3.1. Photos of the investigated sample crystals

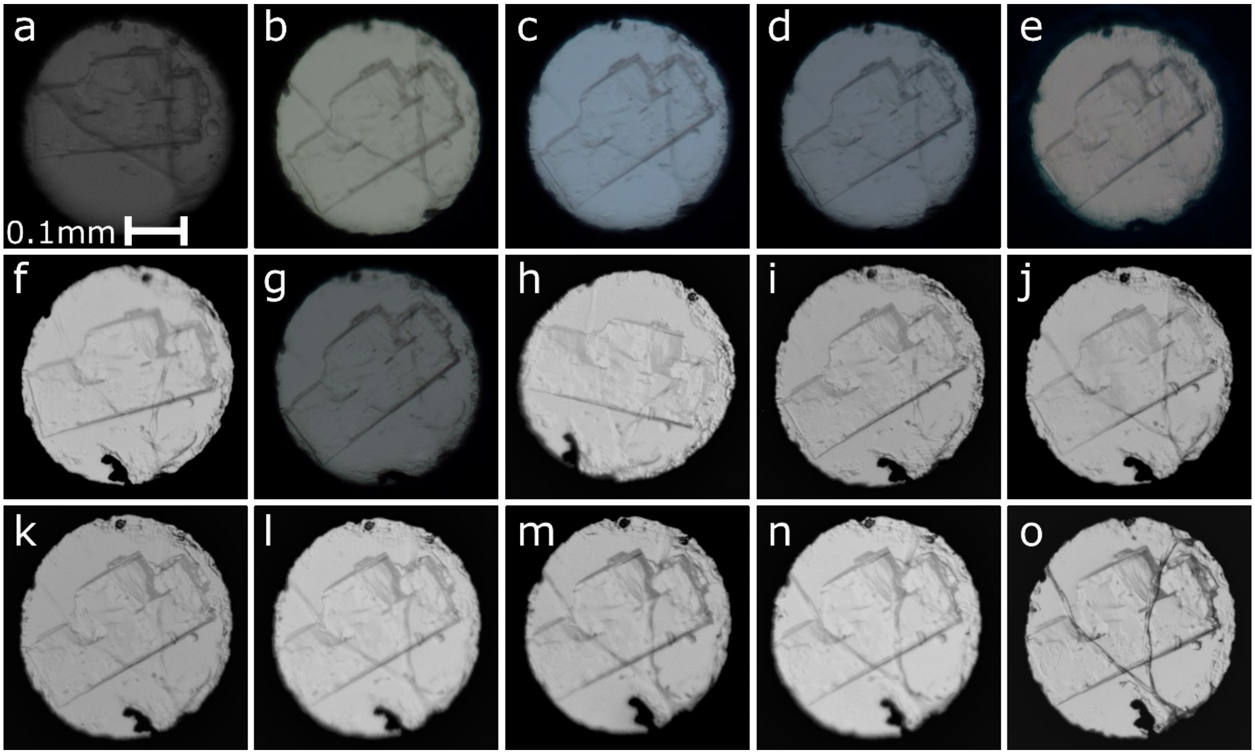

Figure S1. BIPYMA sample crystal at 298 K compressed to (a) 0.25(2); (b) 0.66(2); (c) 1.03(2); (d) 1.32(2); (e) 1.83(2); (f) 1.95(2); (g) 2.19(2); (h) 2.47(2) GPa, as well as at 298 K decompressed to (i) 2.10(2); (j) 1.74(2); (k) 1.54(2); (l) 0.87(2); (m) 0.71(2); (n) 0.48(2); (o) 0.14 GPa. The scale is included in section (a) of the figure. Ruby chip is visible near the gasket edge, and filter fibre holding the crystal is stretched across the opening of the gasket.

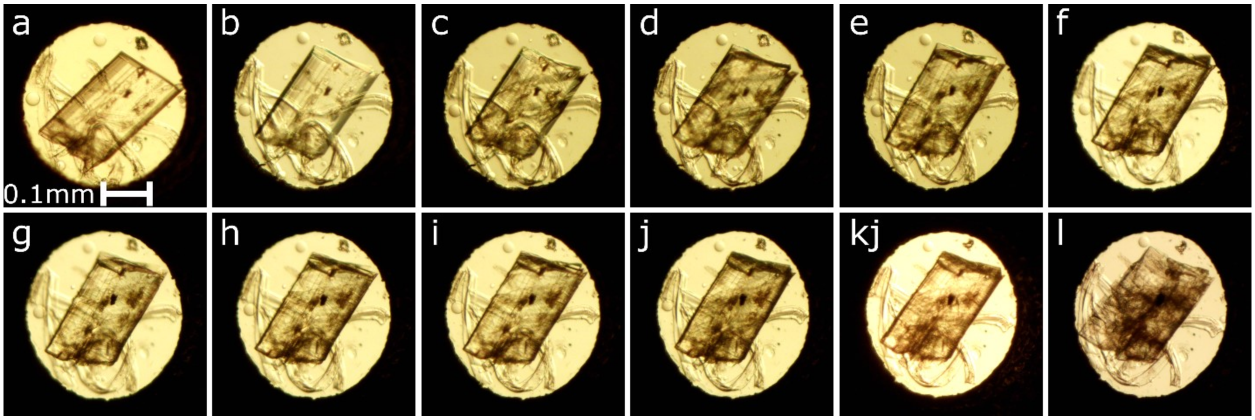

Figure S2. BIPYMA sample crystal at 298 K systematically compressed and decompressed to the pressure in the following order (a) 0.21(2); (b) 2.86(2); (c) 3.37(2); (d) 3.08(2); (e) 2.39(2); (f) 3.35(2); (g) 2.27(2); (h) 3.12(2) GPa; (i) 2.35(2); (j) 3.56(2); (k) 2.40(2); (l) 2.04(2) GPa. At 2.86(2), 2.39(3) and 2.40(2) GPa unit-cell parameters were measured showing that in two first cases crystal had $C2/c$ symmetry, and the last one $P2_1/c$ symmetry. Gradual deterioration of the sample crystal with each compression/decompression run is visible, with final destruction of the crystal shown in section (l). The scale is included in section (a) of the figure. Ruby chip is visible above the sample crystal, and filter fibre holding the crystal is stretched across the opening of the gasket.

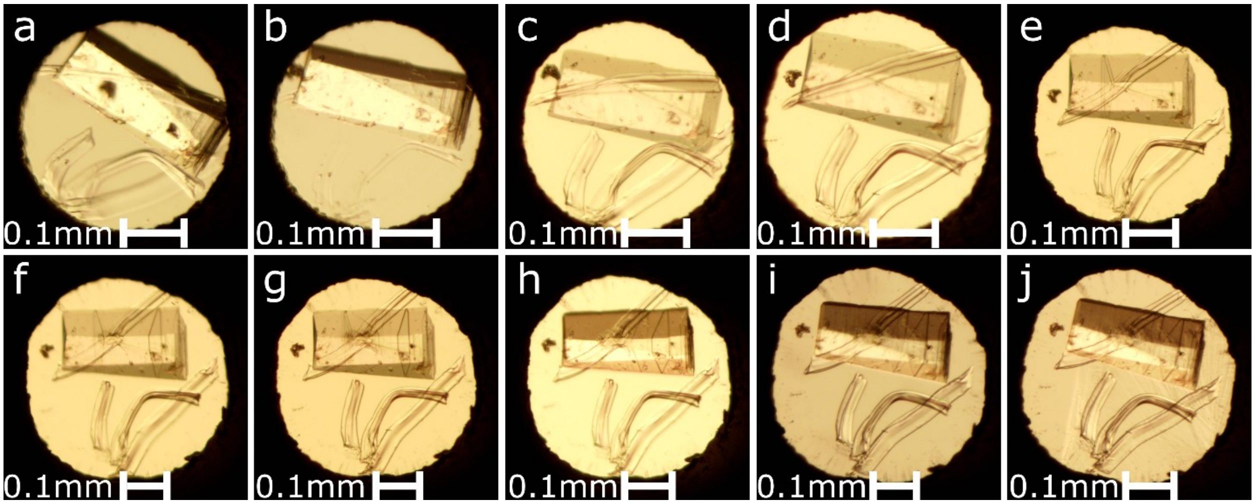

Figure S3. BIPYMA sample crystal at 298 K just after closing the DAC (a), and compressed to (b) 0.25(2); (c) 1.01(2); (d) 1.84(2); (e) 2.62(2); (f) 2.98(2); (g) 3.12(2); (h) 3.17(2); (i) 3.22(2) GPa, as well as at 298 K decompressed to (j) 2.76(2) GPa. Ruby chip is visible to the left of the crystal, and filter fibre holding the crystal is stretched across the opening of the gasket. During compression the opening of the gasket changed significantly therefore the scale was included for each figure section separately.

S3.2. Raman spectra

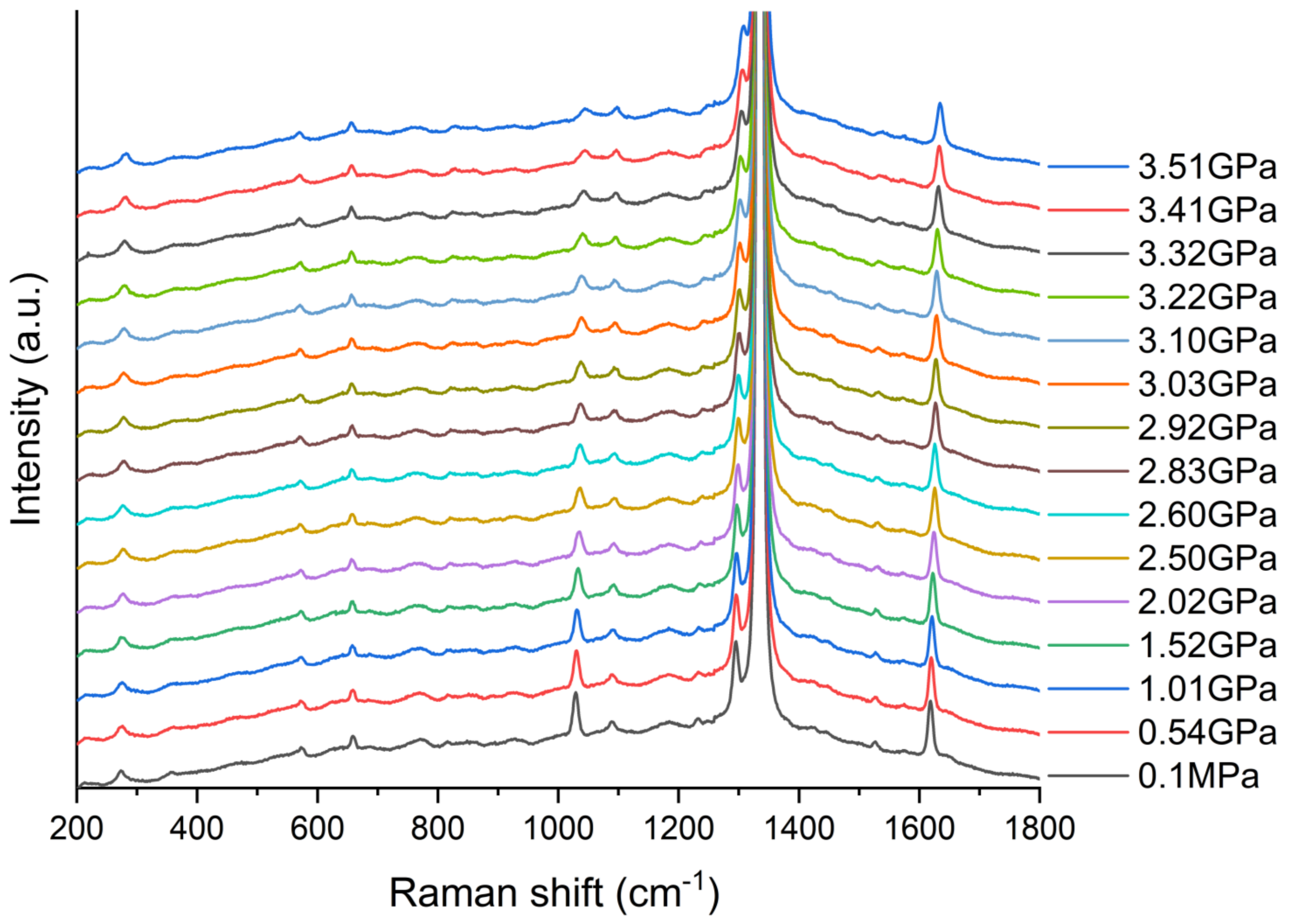

Figure S4. Raman spectra for gradual sample compression. Spectra were measured in order, from bottom to top.

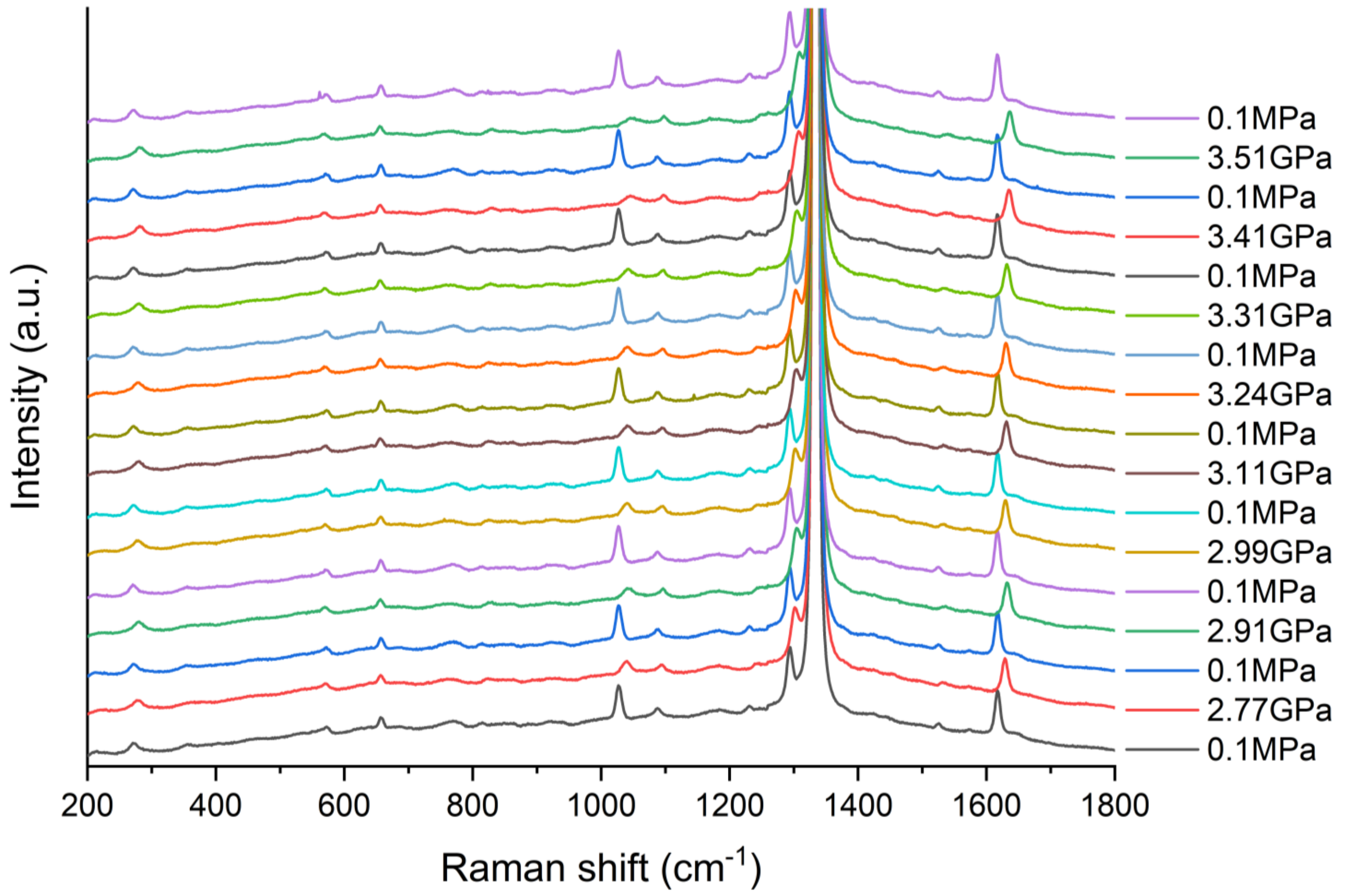

Figure S5. Raman spectra for rapid sample compression (with spectra recorded for decompressed sample after each step). Spectra were measured in order from bottom to top.

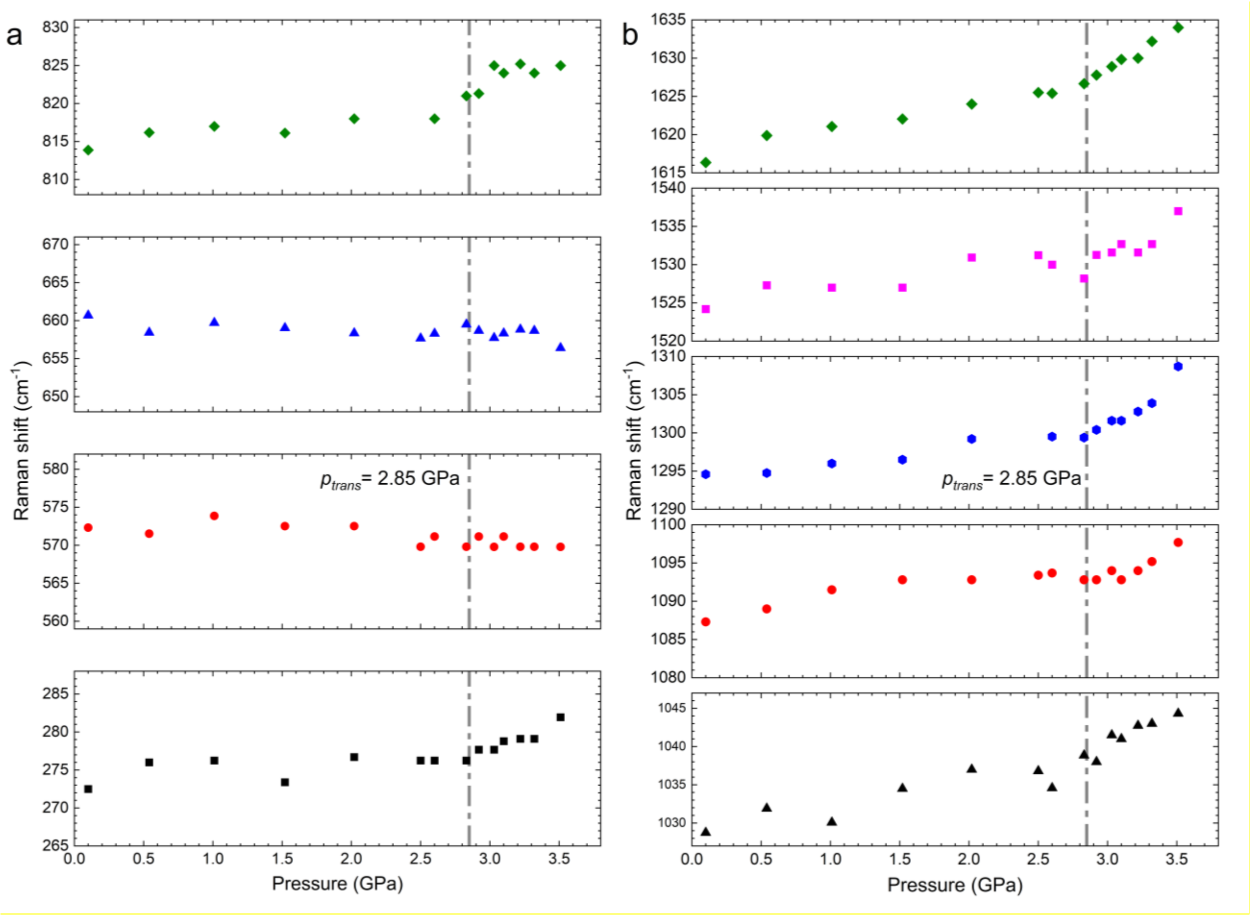

Figure S6. Experimental frequency-pressure data of the main modes between 250 and 830 cm^{-1} (a), as well as 1000 and 1650 cm^{-1} (b). At around 2.85 GPa a slope change is observed.

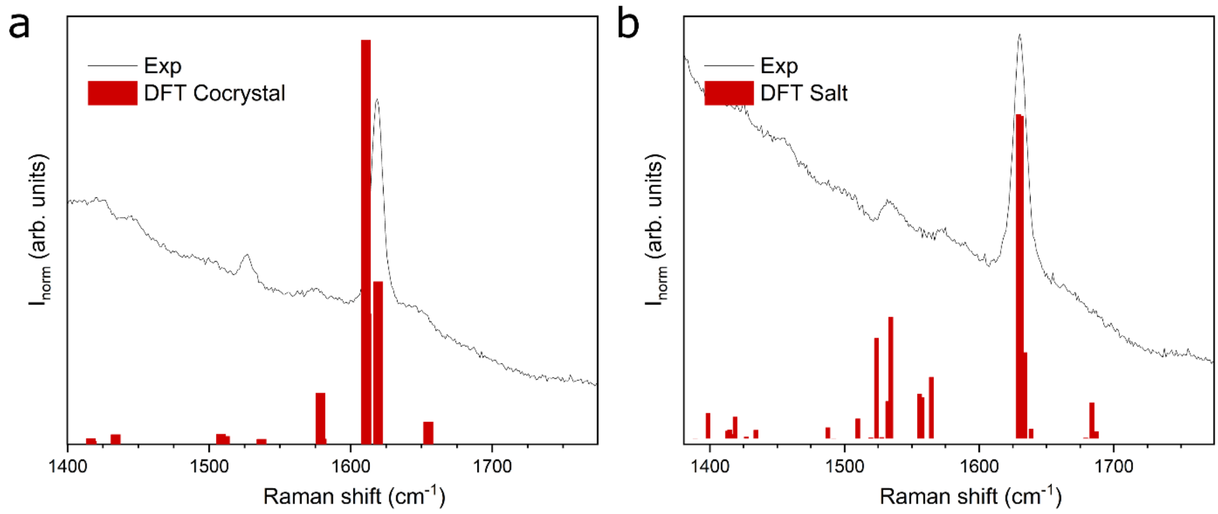

Figure S7. Comparison of Raman modes from experiment and simulations for (a) cocrystal at 0 GPa and (b) salt at 3.14 GPa.

S3.3. Bond lengths and interatomic distances from DFT calculations

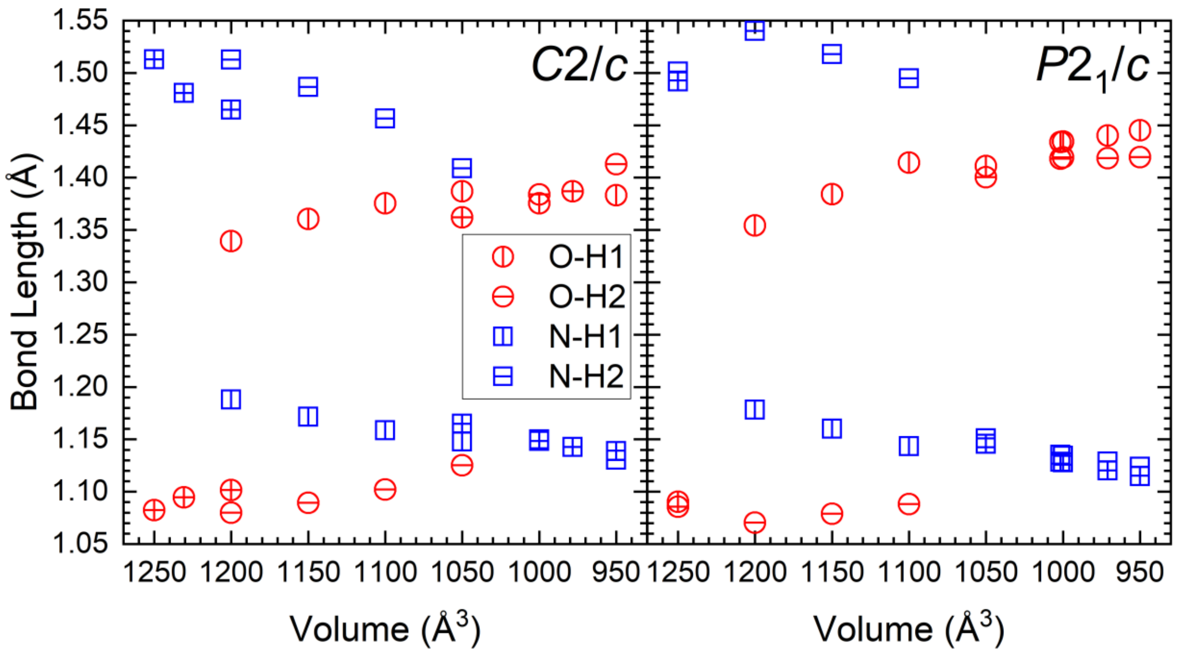

Figure S8. The O-H and N-H bond lengths, shown in red and blue, respectively, obtained from DFT calculations when all atoms in the MA and BIPY molecules were treated as symmetry-independent. Results of calculations in $C2/c$ space group are shown on the left and in $P2_1/c$ on the right.

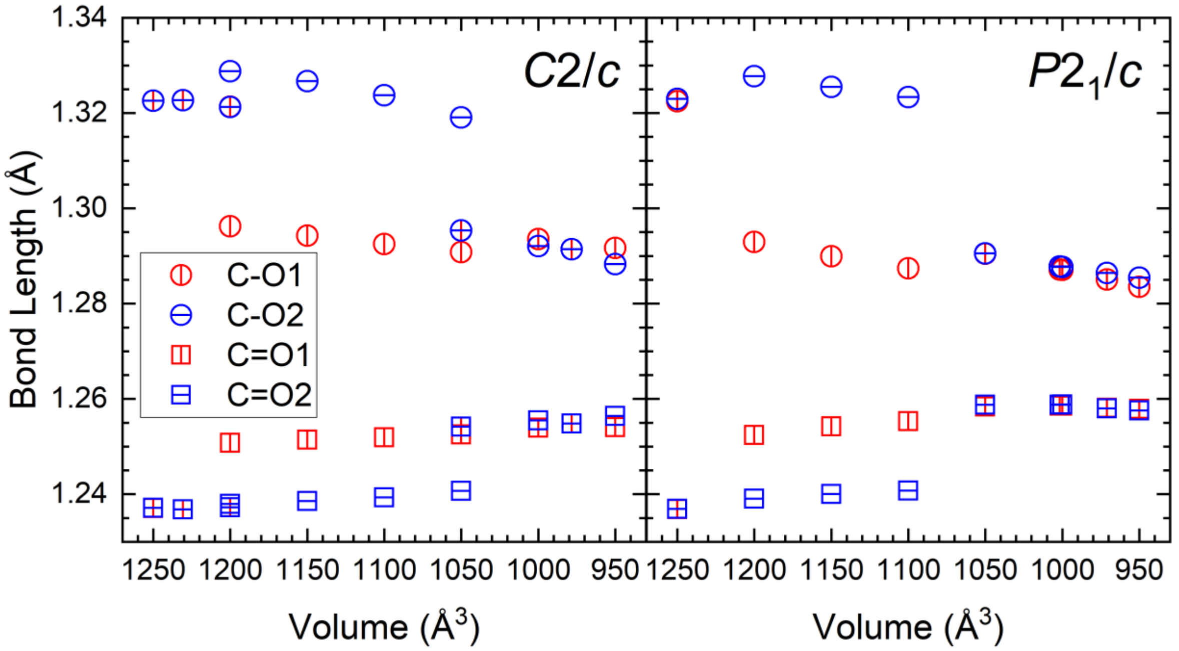

Figure S9. The C-O and C=O bond lengths, shown in red and blue, respectively, obtained from DFT calculations when all atoms in the MA and BIPY molecules were treated as symmetry-independent. Results of calculations in $C2/c$ space group are shown on the left and in $P2_1/c$ on the right.

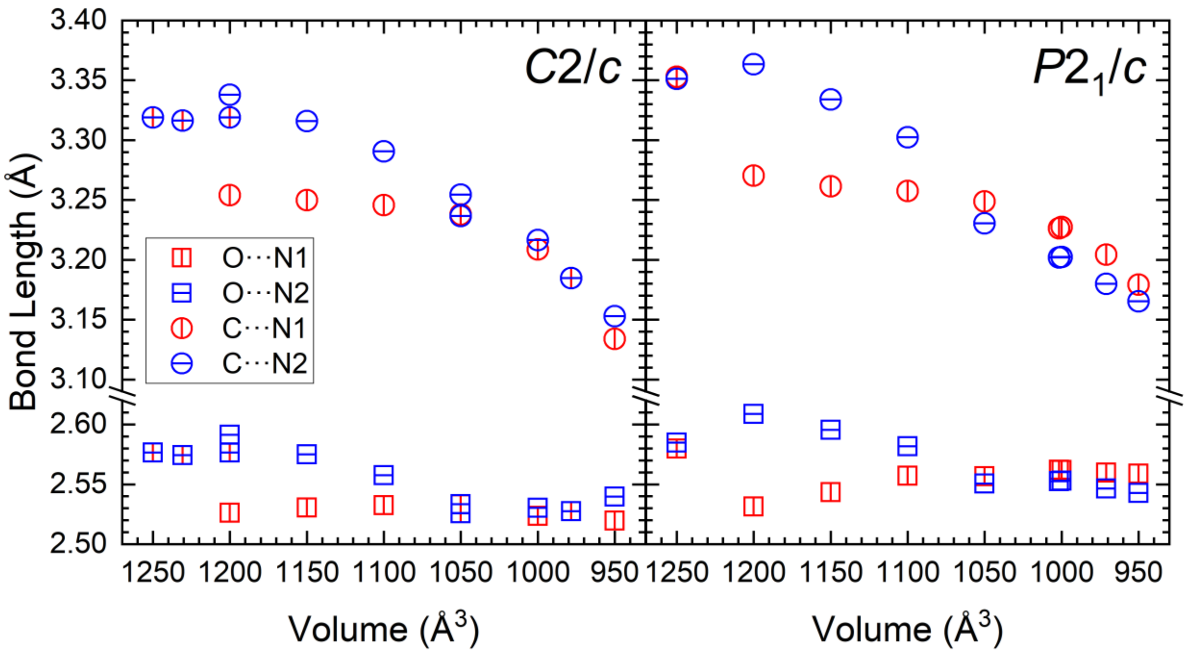

Figure S10. The O···N and C···N distances, shown in red and blue, respectively, obtained from DFT calculations when all atoms in the MA and BIPY molecules were treated as symmetry-independent. Results of calculations in $C2/c$ space group are shown on the left and in $P2_1/c$ on the right.

S3.4. DFT energies

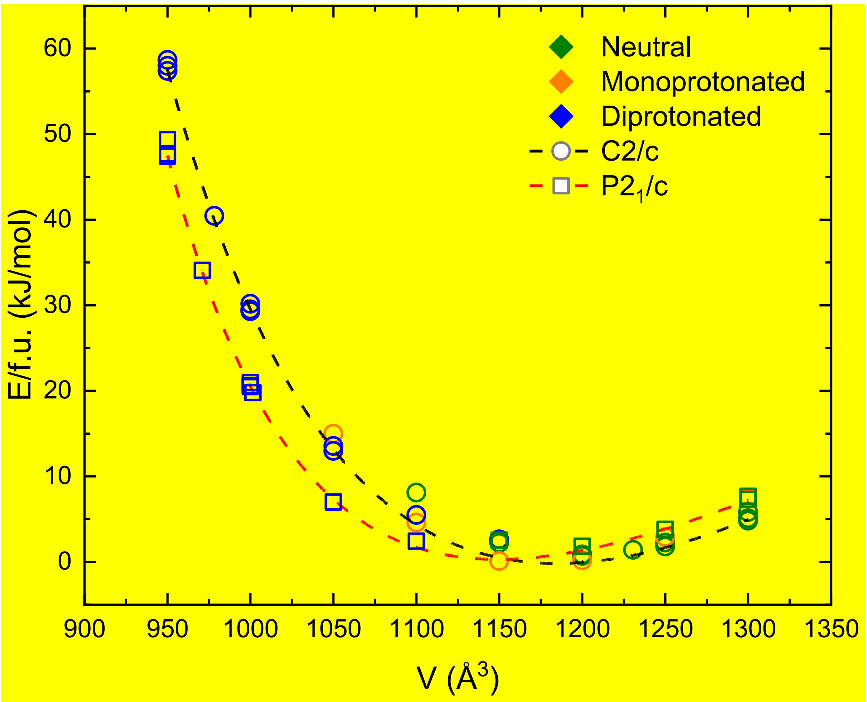

Figure S11. Energy vs Volume points of all the structures computed. Colours correspond to the protonation state after optimisation (green - neutral BIPY, orange - monoprotonated BIPY, blue - diprotonated BIPY).

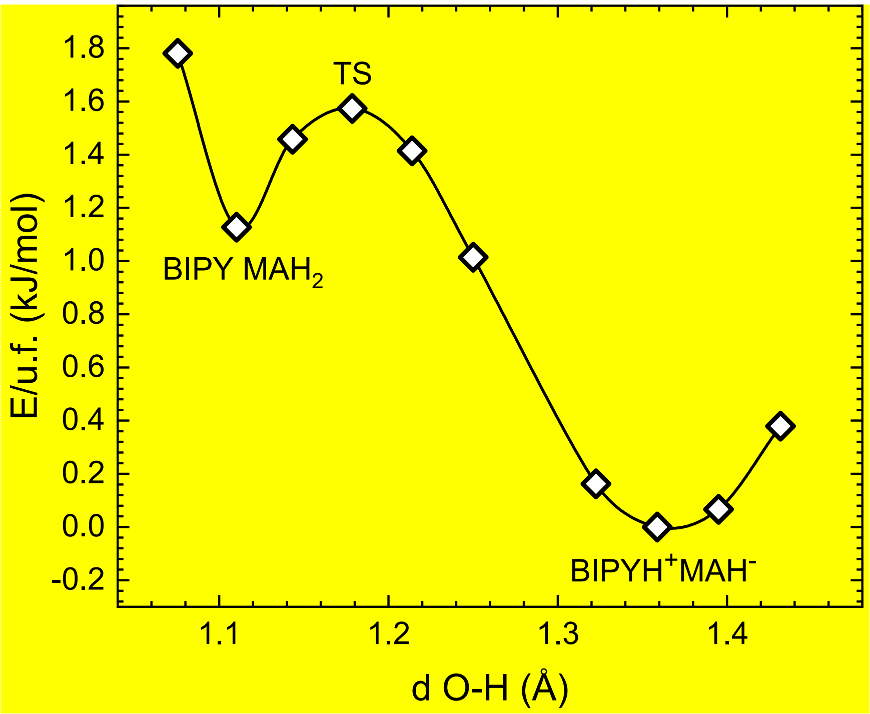

Figure S12. Energy barrier for the proton transfer in the $C2/c$ space group at 1150 Å^3.

S4. Proton-transfer induced changes in aggregation motifs

The phase transition and release of the repulsing interactions between $BIPYH_2^{2+}$ ions affect the geometry of the main H-bonded motif in the structure, a zigzag chain built alternately from MA and BIPY entities (Figure S4), propagating approximately in [40-3] and [30-5] directions in *C*2/*c* and $P2_1/c$ phases, respectively. On compression to 2.5 GPa, the N···C···N and C···C···C angles remain almost unaffected (Figures S13 and S14), the first one decreasing about 0.4° when comparing values at ambient pressure and 2.5 GPa, with the value oscillating within the 113.2(2)-111.99(14)° range for all *C*2/*c* phase structures. Meanwhile, the second angle increases about 1.4° (from 118.05(5) at 0.1 MPa to 119.45(10) at 2.5 GPa). It is visible for the superimposed structures (Figure S13) that compression up to 2.5 has hardly any effect on the structure of the zigzag chain formed by the MA and BIPY.

The double proton transfer reaction leads to decrease in both N···C···N and C···C···C angles, to 106.0(3) and 103.46(13)° at 3.33 GPa, respectively, which results in a geometry of the zigzag chain in salt crystals significantly different compared to the *C*2/*c* phase (Figure S13). On decompression to 2.76 GPa, the N···C···N and C···C···C angles in $P2_1/c$ phase increase by 0.8 and 0.28°, respectively.

To assess the overall compression of the chain formed by BIPYA and MA molecules/ions, the distance between the nitrogen atom of pyridyl group and carbon atom of the carboxylic group should be considered. As the O···N distance is affected by the changes in the length of the C─OH bond that becomes shorter upon deprotonation the bonds between carbon and oxygen atoms become delocalized (the change from ca 1.301(2) Å to 1.25(2) Å between structures at 0.1 MPa and 3.04 GPa). Indeed, when this distance is analysed, it is evident that molecules/ions become closer to each other on the compression to 2.47 GPa, however the significant decrease is observed after phase transition, with the distance being almost the same on both sides of the MA^- ion at 3.04 and 3.33 GPa (the drop from 3.220(12) Å at 2.47 GPa to 3.144(16)/3.153(17) Å, and to 3.15(2)/3.14(2) Å, respectively). Similarly, stronger decrease in the C···N distance was observed in DFT calculations (Figure S10). On decompression from 3.33 GPa to 2.76 GPa those distances become longer and closer in length to those in *C*2/*c* phase at 2.47 GPa.

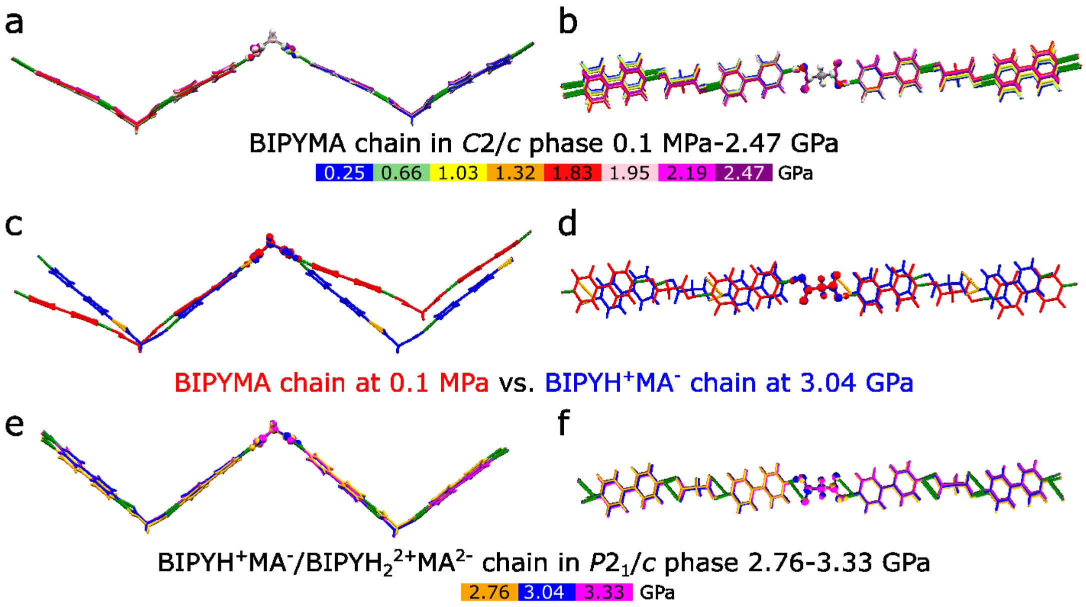

Figure S13. Fragment of the MA···BIPY chains (with central MA molecule/ion superimposed) in $C2/c$ phase in 0.1 MPa-2.47 GPa pressure range (a,b), and in $P2_1/c$ phase in 2.76-3.33 GPa range (e,f). The comparison of MA···BIPY chain in $C2/c$ phase at 0.1 MPa and MA^-···$BIPYH^+$ chain in $P2_1/c$ phase at 3.04 GPa is shown in sections (c) and (d).

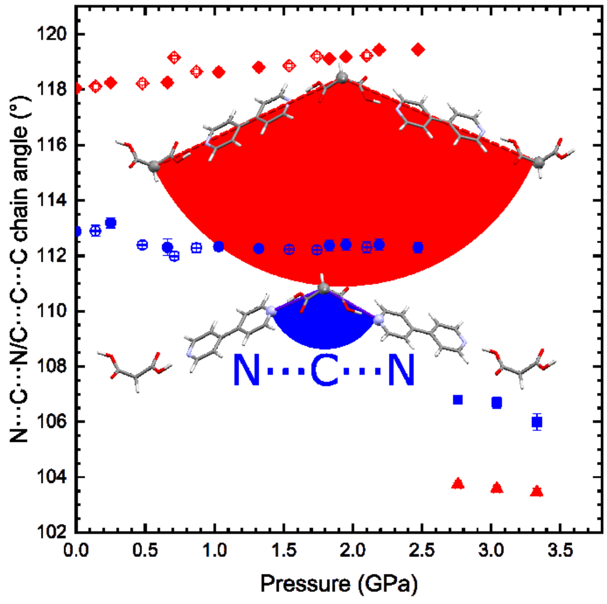

Figure S14. Pressure dependence of N···C···N and C···C···C angles of $BIPYMA/BIPYH^+MA^-/BIPYH_2^{2+}MA^{2-}$chains shown in blue and red, respectively. Data for $C2/c$ phase are shown with circles and diamonds, and for $P2_1/c$ phase with squares and triangles. Empty symbols mark data for structures od decompressed crystal. The insert shows the manner each angle was measured.

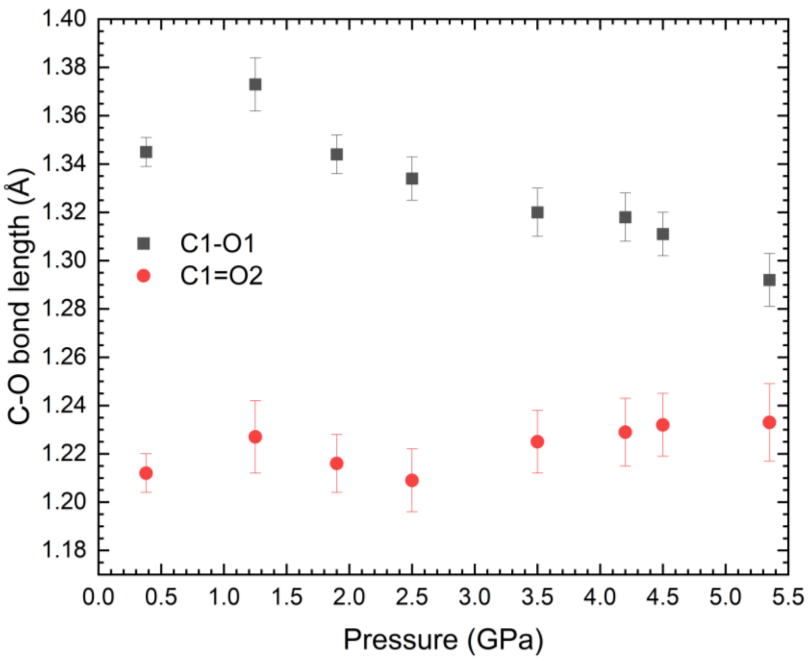

Figure S15. Pressure dependence of the carbon-oxygen bond lengths in crystal structure of PYSUC. Authors of the original report did not provide the pressure values for each structure in the paper and in the CIFs, and therefore the pressure values were deduced from the Figure 3 of the original manuscript (Ward *et al.*, 2023) assuming the numbering in the names of the structures reflect the order of the measurements on increasing the pressure.

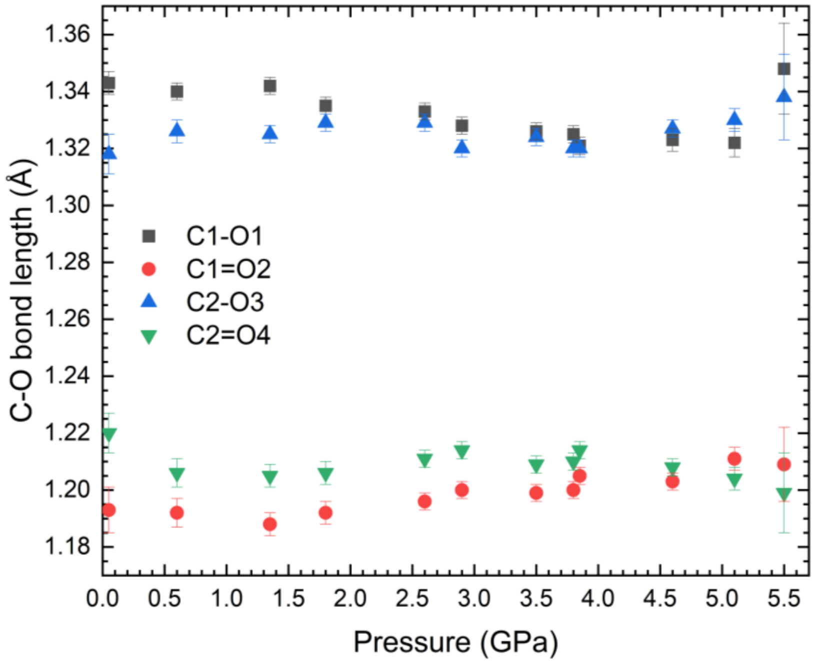

Figure S16. Pressure dependence of the carbon-oxygen bond lengths in crystal structure of PYGLU. Authors of the original report did not provide the pressure values for each structure in the paper and in the CIFs, and therefore the pressure values were deduced from the Figure 3 of the original manuscript (Ward *et al.*, 2023) assuming the numbering in the names of the structures reflect the order of the measurements on increasing the pressure.

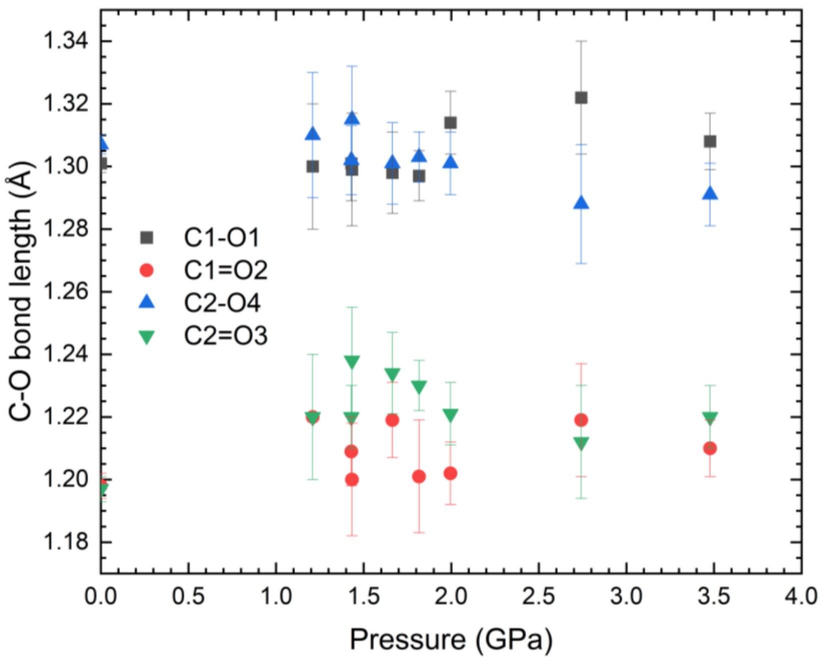

Figure S17. Pressure dependence of the carbon-oxygen bond lengths in crystal structure of PYOX.

S5. References

Abe, H., Imai, Y., Takekiyo, T., Yoshimura, Y. & Hamaya, N. (2014). *IOP Conf. Ser.: Mater. Sci. Eng.* **54**, 012003.

Andrzejewski, M., Olejniczak, A. & Katrusiak, A. (2011). *Crystal Growth & Design* **11**, 4892–4899.

Andrzejewski, M., Olejniczak, A. & Katrusiak, A. (2012). *CrystEngComm* **14**, 6374-6434.

Anioła, M. & Katrusiak, A. (2016). *CrystEngComm* **18**, 3223–3228.

Anioła, M. & Katrusiak, A. (2017). *Crystal Growth & Design* **17**, 3134–3141.

Anioła, M., Kwaśna, K., Cai, W. & Katrusiak, A. (2016). *Crystal Growth & Design* **16**, 6304–6309.

Bąkowicz, J. & Turowska-Tyrk, I. (2016). *CrystEngComm* **18**, 8898–8905.

Bąkowicz, J. & Turowska-Tyrk, I. (2020). *Crystals* **10**, 1031.

Bąkowicz, J. & Turowska-Tyrk, I. (2022). *Acta Crystallogr B Struct Sci Cryst Eng Mater* **78**, 223–230.

Bedeković, N., Stilinović, V., Friščić, T. & Cinčić, D. (2018). *New J. Chem.* **42**, 10584–10591.

Bezzu, C. G., Burt, L. A., McMonagle, C. J., Moggach, S. A., Kariuki, B. M., Allan, D. R., Warren, M. & McKeown, N. B. (2019). *Nat. Mater.* **18**, 740–745.

Bhatt, H., Mishra, A. K., Murli, C., Verma, A. K., Garg, N., Deo, M. N. & Sharma, S. M. (2016). *Phys. Chem. Chem. Phys.* **18**, 8065–8074.

Bogdanov, N. E., Korabel'nikov, D. V., Fedorov, I. A., Zakharov, B. A. & Boldyreva, E. V. (2022). *Acta Crystallogr B Struct Sci Cryst Eng Mater* **78**, 756–762.

Bogdanov, N. E., Milašinović, V., Zakharov, B. A., Boldyreva, E. V. & Molčanov, K. (2020). *Acta Crystallogr B Struct Sci Cryst Eng Mater* **76**, 285–291.

Boschmann, E. & Miller, R. D. (2018). *Tetrahedron* **74**, 2617–2619.

Budzianowski, A. & Katrusiak, A. (2006). *J. Phys. Chem. B* **110**, 9755–9758.

Bujak, M. & Angel, R. J. (2006). *J. Phys. Chem. B* **110**, 10322–10331.

Cameron, C. A., Allan, D. R., Kamenev, K. V., Moggach, S. A., Murrie, M. & Parsons, S. (2014). *Zeitschrift Für Kristallographie – Crystalline Materials* **229**, https://doi.org/10.1515/zkri-2013-1688.

Carlsson, S., Zorina, L., Allan, D. R., Attfield, J. P., Canadell, E. & Batail, P. (2013). *Inorg. Chem.* **52**, 3326–3333.

Casati, N., Macchi, P. & Sironi, A. (2009). *Chem. Commun.* 2679–2681.

Chasák, J., Šlachtová, V., Urban, M. & Brulíková, L. (2021). *European Journal of Medicinal Chemistry* **209**, 112872.

Chemicalize ChemAxon.

Chia, A. S. & Trimble, R. F. Jr. (1961). *J. Phys. Chem.* **65**, 863–866.

Collings, I. E. & Hanfland, M. (2019). *Molecules* **24**, 1759.

Connor, L. E., Delori, A., Hutchison, I. B., Nic Daeid, N., Sutcliffe, O. B. & Oswald, I. D. H. (2015). *Acta Crystallogr B Struct Sci Cryst Eng Mater* **71**, 3–9.

Craig, G. A., Sarkar, A., Woodall, C. H., Hay, M. A., Marriott, K. E. R., Kamenev, K. V., Moggach, S. A., Brechin, E. K., Parsons, S., Rajaraman, G. & Murrie, M. (2018). *Chem. Sci.* **9**, 1551–1559.

Dey, B. P. & Lahiri, S. C. (2010). *Journal of the Indian Chemical Society* **87**, 29–41.

Eikeland, E., Thomsen, M. K., Madsen, S. R., Overgaard, J., Spackman, M. A. & Iversen, Bo. B. (2016). *Chemistry – A European Journal* **22**, 4061–4069.

Eikeland, E., Thomsen, M. K., Overgaard, J., Spackman, M. A. & Iversen, B. B. (2017). *Crystal Growth & Design* **17**, 3834–3846.

Eikeland, E. Z., Borup, M., Thomsen, M. K., Roelsgaard, M., Overgaard, J., Spackman, M. A. & Iversen, B. B. (2020). *Crystal Growth & Design* **20**, 4092–4099.

Fabbiani, F. P. A., Allan, D. R., David, W. I. F., Davidson, A. J., Lennie, A. R., Parsons, S., Pulham, C. R. & Warren, J. E. (2007). *Crystal Growth & Design* **7**, 1115–1124.

Fabbiani, F. P. A., Allan, D. R., David, W. I. F., Moggach, S. A., Parsons, S. & Pulham, C. R. (2004). *CrystEngComm* **6**, 504–511.

Fabbiani, F. P. A., Allan, D. R., Dawson, A., David, W. I. F., McGregor, P. A., Oswald, I. D. H., Parsons, S. & Pulham, C. R. (2003). *Chem. Commun.* 3004-3005.

Fabbiani, F. P. A., Bergantin, S., Gavezzotti, A., Rizzato, S. & Moret, M. (2016). *CrystEngComm* **18**, 2173–2181.

Fabbiani, F. P. A., Buth, G., Levendis, D. C. & Cruz-Cabeza, A. J. (2014). *Chem. Commun.* **50**, 1817–1819.

Fabbiani, F. P. A., Dittrich, B., Florence, A. J., Gelbrich, T., Hursthouse, M. B., Kuhs, W. F., Shankland, N. & Sowa, H. (2009). *CrystEngComm* **11**, 1396-1406.

Fabbiani, F. P. A., Levendis, D. C., Buth, G., Kuhs, W. F., Shankland, N. & Sowa, H. (2010). *CrystEngComm* **12**, 2354-2360.

Filhol, A., Bravic, G., Gaultier, J., Chasseau, D. & Vettier, C. (1981). *Acta Crystallogr B Struct Crystallogr Cryst Chem* **37**, 1225–1235.

Fornasari, L., Olejniczak, A., Rossi, F., d'Agostino, S., Chierotti, M. R., Gobetto, R., Katrusiak, A. & Braga, D. (2020). *Chem. Eur. J.* **26**, 5061–5069.

Friedrich, A., Collings, I. E., Dziubek, K. F., Fanetti, S., Radacki, K., Ruiz-Fuertes, J., Pellicer-Porres, J., Hanfland, M., Sieh, D., Bini, R., Clark, S. J. & Marder, T. B. (2020). *J. Am. Chem. Soc.* **142**, 18907–18923.

Funnell, N. P., Allan, D. R., Maloney, A. G. P., Smith, R. I., Wilson, C. J. G. & Parsons, S. (2021). *CrystEngComm* **23**, 769–776.

Galica, T., Konieczny, K. A. & Turowska-Tyrk, I. (2020). *Journal of Photochemistry and Photobiology A: Chemistry* **386**, 112119.

Galica, T. & Turowska-Tyrk, I. (2019). *Journal of Photochemistry and Photobiology A: Chemistry* **376**, 108–115.

Galiois, B., Gaultier, J., Hauw, C., Chasseau, D., Meresse, A., Filhol, A. & Bechgaard, K. (1985). *Molecular Crystals and Liquid Crystals* **119**, 225–232.

Gallois, B., Gaultier, J., Bechtel, F., Filhol, A. & Vettier, C. (1987). *Molecular Crystals and Liquid Crystals* **148**, 279–293.

Gallois, B., Gaultier, J., Hauw, C., Lamcharfi, T. -d. & Filhol, A. (1986). *Acta Crystallogr B Struct Sci* **42**, 564–575.

Galloway, K. W., Moggach, S. A., Parois, P., Lennie, A. R., Warren, J. E., Brechin, E. K., Peacock, R. D., Valiente, R., González, J., Rodríguez, F., Parsons, S. & Murrie, M. (2010). *CrystEngComm* **12**, 2516.

G. Ehrenreich, M., Zeng, Z., Burger, S., R. Warren, M., W. Gaultois, M., Tan, J.-C. & Kieslich, G. (2019). *Chemical Communications* **55**, 3911–3914.

Giordano, N., Beavers, C. M., Kamenev, K. V., Love, J. B., Pankhurst, J. R., Teat, S. J. & Parsons, S. (2020). *Chem. Commun.* **56**, 3449–3452.

Granero-García, R., Falenty, A. & Fabbiani, F. P. A. (2017). *Chemistry A European J* **23**, 3691–3698.

Guionneau, P., Brigouleix, C., Barrans, Y., Goeta, A. E., Létard, J.-F., Howard, J. A. K., Gaultier, J. & Chasseau, D. (2001). *Comptes Rendus de l'Académie Des Sciences - Series IIC - Chemistry* **4**, 161–171.

Guionneau, P., Gaultier, J., Rahal, M., Bravic, G., M. Mellado, J., Chasseau, D., Ducasse, L., Kurmoo, M. & Day, P. (1995). *Journal of Materials Chemistry* **5**, 1639–1645.

Guranda, D. T., Ushakov, G. A., Yolkin, P. G. & Švedas, V. K. (2012). *Journal of Molecular Catalysis B: Enzymatic* **74**, 48–53.

Horiuchi, S., Kumai, R. & Tokura, Y. (2013). *J. Am. Chem. Soc.* **135**, 4492–4500.

H. Oswald, I. D. & R. Pulham, C. (2008). *CrystEngComm* **10**, 1114–1116.

Huq, A. & Stephens, P. W. (2006). *Phys. Rev. B* **74**, 075424.

Jaroń, T., Starobrat, A., Struzhkin, V. V. & Grochala, W. (2020). *Eur. J. Inorg. Chem.* **2020**, 3846–3851.

Johnstone, R. D. L., Francis, D., Lennie, A. R., Marshall, W. G., Moggach, S. A., Parsons, S., Pidcock, E. & Warren, J. E. (2008). *CrystEngComm* **10**, 1758.

Johnstone, R. D. L., Lennie, A. R., Parsons, S., Pidcock, E. & Warren, J. E. (2009). *Acta Cryst B* **65**, 731–748.

Jungen, S., Paenurk, E. & Chen, P. (2020). *Inorg. Chem.* **59**, 12322–12336.

Katrusiak, A., Szafrański, M. & Podsiadło, M. (2011). *Chem. Commun.* **47**, 2107–2109.

Keller, S., Prescimone, A., Bolink, H., Sessolo, M., Longo, G., Martínez-Sarti, L., Junquera-Hernández, J. M., Constable, E. C., Ortí, E. & Housecroft, C. E. (2018). *Dalton Trans.* **47**, 14263–14276.

Keller, S., Prescimone, A., Constable, E. C. & Housecroft, C. E. (2018). *Photochem Photobiol Sci* **17**, 375–385.

Khalil, M. M., Radalla, A. M. & Abd Elnaby, N. M. (2013). *J Solution Chem* **42**, 1123–1145.

Khalili, F., Henni, A. & East, A. L. L. (2009). *J. Chem. Eng. Data* **54**, 2914–2917.

Komatsu, K., Machida, S., Noritake, F., Hattori, T., Sano-Furukawa, A., Yamane, R., Yamashita, K. & Kagi, H. (2020). *Nat Commun* **11**, 464.

Konieczny, K. A., Bąkowicz, J., Paliwoda, D., Warren, M. R., Ciesielski, A., Cyrański, M. K. & Turowska-Tyrk, I. (2021). *Acta Crystallographica Section B* **77**, 321–330.

Konieczny, K. A., Szczurek, A., Bąkowicz, J., Siedlecka, R., Ciesielski, A., Cyrański, M. K. & Turowska-Tyrk, I. (2020). *Crystal Growth & Design* **20**, 5061–5071.

Konieczny, K., Bąkowicz, J., Galica, T., Siedlecka, R. & Turowska-Tyrk, I. (2017). *CrystEngComm* **19**, 3044–3050.

Konieczny, K., Bąkowicz, J. & Turowska-Tyrk, I. (2015). *CrystEngComm* **17**, 7693–7701.

Konieczny, K., Bąkowicz, J. & Turowska-Tyrk, I. (2016). *Journal of Photochemistry and Photobiology A: Chemistry* **325**, 111–115.

Konieczny, K., Ciesielski, A., Bąkowicz, J., Galica, T. & Turowska-Tyrk, I. (2018). *Crystals* **8**, 299.

Kortüm, G., Vogel, W. & Andrussow, K. Dissociation Constants of Organic Acids in Aqueous Solution De Gruyter.

Kozlenko, D. P., Wasicki, J. W., Glazkov, V. P., Kichanov, S. E., Nawrocik, W. & Savenko, B. N. (2005). *Crystallogr. Rep.* **50**, 78–84.

Kumai, R., Horiuchi, S., Fujioka, J. & Tokura, Y. (2012). *J. Am. Chem. Soc.* **134**, 1036–1046.

Kurnosov, A. V., Komarov, V. Yu., Voronin, V. I., Teplykh, A. E. & Manakov, A. Yu. (2004). *Angewandte Chemie International Edition* **43**, 2922–2924.

Le Pevelen, D., Barrans, Y., Gaultier, J. & Chasseau, D. (1999). *Synthetic Metals* **102**, 1609–1610.

LeBlanc, L. M., Dale, S. G., Taylor, C. R., Becke, A. D., Day, G. M. & Johnson, E. R. (2018). *Angew Chem Int Ed* **57**, 14906–14910.

Losev, E. A., Zakharov, B. A. & Boldyreva, E. V. (2016). *CrystEngComm* **18**, 5869–5875.

Lugo, M. L. & Lubes, V. R. (2007). *J. Chem. Eng. Data* **52**, 1217–1222.

Ma, Z., Li, J., Liu, C., Sun, C. & Zhou, M. (2017). *Sci Rep* **7**, 4677.

Macchi, P., Casati, N., Marshall, W. G. & Sironi, A. (2010). *CrystEngComm* **12**, 2596–2603.

Madsen, S. R., Thomsen, M. K., Scheins, S., Chen, Y.-S., Finkelmeier, N., Stalke, D., Overgaard, J. & Iversen, B. B. (2014). *Dalton Trans.* **43**, 1313–1320.

Mailman, A., Wong, J. W. L., Winter, S. M., Claridge, R. C. M., Robertson, C. M., Assoud, A., Yong, W., Steven, E., Dube, P. A., Tse, J. S., Desgreniers, S., Secco, R. A. & Oakley, R. T. (2017). *J. Am. Chem. Soc.* **139**, 1625–1635.

Milašinović, V., Molčanov, K., Krawczuk, A., Bogdanov, N. E., Zakharov, B. A., Boldyreva, E. V., Jelsch, C. & Kojić-Prodić, B. (2021). *IUCrJ* **8**, 644–654.

Mínguez Espallargas, G., Brammer, L., Allan, D. R., Pulham, C. R., Robertson, N. & Warren, J. E. (2008). *J. Am. Chem. Soc.* **130**, 9058–9071.

Moggach, S. A., Galloway, K. W., Lennie, A. R., Parois, P., Rowantree, N., Brechin, E. K., Warren, J. E., Murrie, M. & Parsons, S. (2009). *CrystEngComm* **11**, 2601-2604.

Morency, M., Néron, S., Iftimie, R. & Wuest, J. D. (2021). *J. Org. Chem.* **86**, 14444–14460.

Nam, T., Nara, S. J., Zagol-Ikapitte, I., Cooper, T., Valgimigli, L., Oates, J. A., Porter, N. A., Boutaud, O. & Pratt, D. A. (2009). *Org. Biomol. Chem.* **7**, 5103–5112.

Naumov, P., Sahoo, S. C., Zakharov, B. A. & Boldyreva, E. V. (2013). *Angew. Chem. Int. Ed.* **52**, 9990–9995.

Nazeeruddin, M. K. & Kalyanasundaram, K. (1989). *Inorg. Chem.* **28**, 4251–4259.

Nicholas, A. D., Zhao, J., Slebodnick, C., Ross, N. L. & Cahill, C. L. (2021). *Journal of Solid State Chemistry* **300**, 122262.

Nowicki, W., Olejniczak, A., Andrzejewski, M. & Katrusiak, A. (2012). *CrystEngComm* **14**, 6428-6434.

Olejniczak, A. & Katrusiak, A. (2010). *CrystEngComm* **12**, 2528-2532.

Olejniczak, A. & Katrusiak, A. (2011). *Crystal Growth & Design* **11**, 2250–2256.

Olejniczak, A., Katrusiak, A., Podsiadło, M. & Katrusiak, A. (2020). *Acta Crystallogr B Struct Sci Cryst Eng Mater* **76**, 1136–1142.

Olejniczak, A., Katrusiak, A., Podsiadło, M. & Katrusiak, A. (2022*a*). *Crystal Growth & Design* **22**, 5996–6003.

Olejniczak, A., Katrusiak, A., Podsiadło, M. & Katrusiak, A. (2022*b*). *IUCrJ* **9**, 49–54.

Olejniczak, A., Katrusiak, A. & Szafrański, M. (2010). *Crystal Growth & Design* **10**, 3537–3546.

Olejniczak, A., Katrusiak, A. & Vij, A. (2009). *CrystEngComm* **11**, 1240–1244.

Olejniczak, A., Krūkle-Bērziņa, K. & Katrusiak, A. (2016). *Crystal Growth & Design* **16**, 3756–3762.

Olejniczak, A., Szafrański, M. & Katrusiak, A. (2018). *Crystal Growth & Design* **18**, 6488–6496.

Ono, T., Tsukiyama, Y., Taema, A., Sato, H., Kiyooka, H., Yamaguchi, Y., Nagahashi, A., Nishiyama, M., Akahama, Y., Ozawa, Y., Abe, M. & Hisaeda, Y. (2018). *ChemPhotoChem* **2**, 416–420.

Paliwoda, D., Szafrański, M., Hanfland, M. & Katrusiak, A. (2018). *J. Mater. Chem. C* **6**, 7689–7699.

Parois, P., Moggach, S. A., Sanchez-Benitez, J., Kamenev, K. V., Lennie, A. R., Warren, J. E., Brechin, E. K., Parsons, S. & Murrie, M. (2010). *Chem. Commun.* **46**, 1881–1883.

Patyk-Kaźmierczak, E. & Kaźmierczak, M. (2021). *Crystal Growth & Design* **21**, 6879–6888.

Patyk-Kaźmierczak, E., Warren, M. R., Allan, D. R. & Katrusiak, A. (2017). *Phys. Chem. Chem. Phys.* **19**, 9086–9091.

Pfrunder, M. C., Whittaker, J. J., Parsons, S., Moubaraki, B., Murray, K. S., Moggach, S. A., Sharma, N., Micallef, A. S., Clegg, J. K. & McMurtrie, J. C. (2020). *Chem. Mater.* **32**, 3229–3234.

Podsiadło, M., Jakóbek, K. & Katrusiak, A. (2010). *CrystEngComm* **12**, 2561-2567.

Poręba, T., Ernst, M., Zimmer, D., Macchi, P. & Casati, N. (2019). *Angewandte Chemie International Edition* **58**, 6625–6629.

Poręba, T., Macchi, P. & Casati, N. (2020). *Crystal Growth & Design* **20**, 4375–4386.

Prescimone, A., Sanchez-Benitez, J., Kamenev, K. K., Moggach, S. A., Warren, J. E., Lennie, A. R., Murrie, M., Parsons, S. & Brechin, E. K. (2010). *Dalton Trans.* **39**, 113–123.

Prescimone, A., Sanchez-Benitez, J., Kamenev, K. V., Moggach, S. A., Lennie, A. R., Warren, J. E., Murrie, M., Parsons, S. & Brechin, E. K. (2009). *Dalton Trans.* 7390-7395.

Prescimone, A., Sanchez-Benitez, J., Kamenev, K. V., Warren, J. E., Lennie, A. R., Murrie, M., Parsons, S. & Brechin, E. K. (2010). *Zeitschrift Für Naturforschung B* **65**, 221–230.

Price, A. N., Berryman, V., Ochiai, T., Shephard, J. J., Parsons, S., Kaltsoyannis, N. & Arnold, P. L. (2022). *Nat Commun* **13**, 3931.

Priola, E., Curetti, N., Marabello, D., Andreo, J., Giordana, A., Andreo, L., Benna, P., Cavalcante Freire, P. T., Benzi, P., Operti, L. & Diana, E. (2022). *CrystEngComm* **24**, 2336–2348.

Raghavendra, N. (2022). *Russ. J. Phys. Chem.* **96**, 334–339.

Rejnhardt, P., Drozd, M. & Daszkiewicz, M. (2021). *Acta Crystallogr B Struct Sci Cryst Eng Mater* **77**, 996–1002.

Richardson, J. G., Benjamin, H., Moggach, S. A., Warren, L. R., Warren, M. R., Allan, D. R., Saunders, L. K., Morrison, C. A. & Robertson, N. (2020). *Phys. Chem. Chem. Phys.* **22**, 17668–17676.

Rodríguez-Velamazán, J. A., Fabelo, O., Beavers, C. M., Natividad, E., Evangelisti, M. & Roubeau, O. (2014). *Chem. Eur. J.* **20**, 7956–7961.

Roszak, K. & Katrusiak, A. (2021). *Acta Crystallogr B Struct Sci Cryst Eng Mater* **77**, 449–455.

Saouane, S. & Fabbiani, F. P. A. (2015). *Crystal Growth & Design* **15**, 3875–3884.

Satthaphut, Sutcliffe, & Oswaldiain (2014). *Zeitschrift Für Kristallographie – Crystalline Materials* **229**, https://doi.org/10.1515/zkri-2013-1639.

Schmitz, D., Kalter, M., Dunbar, A. C., Vöst, M., Fischer, A., Batke, K., Eickerling, G., Ruhland, K., Ebad-Allah, J., Kuntscher, C. & Scherer, W. (2020). *Eur J Inorg Chem* **2020**, 79–83.

Schultz, A. J., Wang, H. H., Williams, J. M. & Filhol, Alain. (1986). *J. Am. Chem. Soc.* **108**, 7853–7855.

Shepherd, H. J., Palamarciuc, T., Rosa, P., Guionneau, P., Molnár, G., Létard, J.-F. & Bousseksou, A. (2012). *Angew. Chem. Int. Ed.* **51**, 3910–3914.

Shepherd, H. J., Rosa, P., Vendier, L., Casati, N., Létard, J.-F., Bousseksou, A., Guionneau, P. & Molnár, G. (2012). *Phys. Chem. Chem. Phys.* **14**, 5265-5271.

Shepherd, H., Tonge, G., Hatcher, L., Bryant, M., Knichal, J., Raithby, P., Halcrow, M., Kulmaczewski, R., Gagnon, K. & Teat, S. (2016). *Magnetochemistry* **2**, 9.

Shibaeva, R. P., Kaminskii, V. P. & Yagubskii, E. B. (1985). *Molecular Crystals and Liquid Crystals* **119**, 361–373.

Shimizu, S., Watanabe, N., Kataoka, T., Shoji, T., Abe, N., Morishita, S. & Ichimura, H. (2000). Vol. *Ullmann's Encyclopedia of Industrial Chemistry*, edited by Wiley-VCH. Wiley.

Sobczak, S., Półrolniczak, A., Ratajczyk, P., Cai, W., Gładysiak, A., Nikolayenko, V. I., Castell, D. C., Barbour, L. J. & Katrusiak, A. (2020). *Chem. Commun.* **56**, 4324–4327.

Somayazulu, M. S., Finger, L. W., Hemley, R. J. & Mao, H. K. (1996). *Science* **271**, 1400–1402.

Starkey, R., Norman, J. & Hintze, M. (1986). *J. Chem. Educ.* **63**, 473.

Stevens, C. J., Prescimone, A., Tuna, F., McInnes, E. J. L., Parsons, S., Morrison, C. A., Arnold, P. L. & Love, J. B. (2016). *Inorg. Chem.* **55**, 214–220.

Szafrański, M. (2014). *CrystEngComm* **16**, 6250–6256.

Szafrański, M. (2020). *J. Phys. Chem. C* **124**, 11631–11638.

Szafrański, M. & Ståhl, K. (2016). *Crystal Growth & Design* **16**, 2157–2166.

Terlecki, M., Sobczak, S., Leszczyński, M. K., Katrusiak, A. & Lewiński, J. (2021). *Chemistry A European J* **27**, 13757–13764.

Thiel, A. M., Damgaard-Møller, E. & Overgaard, J. (2020). *Inorg. Chem.* **59**, 1682–1691.

Ulstrup, J., Östman, C. O., Strand, T. G., Stølevik, R., Borch, G. & Craig, J. C. (1969). *Acta Chem. Scand.* **23**, 3091–3109.

Wallenfels, K. & Friedrich, K. (1960). *Chemische Berichte* **93**, 3070–3082.

Ward, M. R. & Oswald, I. D. H. (2019). *CrystEngComm* **21**, 4437–4443.

Ward, M. R., Bull, C. L., Funnell, N. P., Warren, M. R. & Oswald, I. D. H. (2023). *International Journal of Pharmaceutics* **647**, 123514.Wong, H. L. S., Allan, D. R., Champness, N. R., McMaster, J., Schröder, M. & Blake, A. J. (2013). *Angew Chem Int Ed* **52**, 5093–5095.

Woodall, C. H., Craig, G. A., Prescimone, A., Misek, M., Cano, J., Faus, J., Probert, M. R., Parsons, S., Moggach, S., Martínez-Lillo, J., Murrie, M., Kamenev, K. V. & Brechin, E. K. (2016). *Nat Commun* **7**, 13870.

Wu, L.-C., Nielsen, M. B., Bremholm, M., Madsen, S. R., Overgaard, J., Newville, M., Chen, Y.-S. & Iversen, B. B. (2015). *Chem. Commun.* **51**, 8868–8871.

Yamaura, J.-I. & Kato, R. (2002). *Molecular Crystals and Liquid Crystals* **379**, 47–52.

Zakharov, B. A. & Boldyreva, E. V. (2013). *Acta Crystallogr B Struct Sci Cryst Eng Mater* **69**, 271–280.

Zakharov, B. A., Losev, E. A. & Boldyreva, E. V. (2013). *CrystEngComm* **15**, 1693–1697.

Zakharov, B. A., Marchuk, A. S. & Boldyreva, E. V. (2015). *CrystEngComm* **17**, 8812–8816.

Zieliński, W. & Katrusiak, A. (2015). *CrystEngComm* **17**, 5468–5473.

Zielinski, W. & Katrusiak, A. (2016). *CrystEngComm* **18**, 3211–3215.
